# Congruence, but no cascade—Pelagic biodiversity across three trophic levels in Nordic lakes

**DOI:** 10.1002/ece3.6514

**Published:** 2020-07-17

**Authors:** Tom Andersen, Dag O. Hessen, Johnny P. Håll, Maryia Khomich, Marcia Kyle, Markus Lindholm, Serena Rasconi, Birger Skjelbred, Jan‐Erik Thrane, Bjørn Walseng

**Affiliations:** ^1^ Department of Biosciences University of Oslo Oslo Norway; ^2^ Norwegian Institute for Water Research Oslo Norway; ^3^ Nofima AS Ås Norway; ^4^ Oslo Norway; ^5^ Rudolf Steiner University College Oslo Norway; ^6^ WasserCluster – Biological Station Lunz Inter‐University Centre for Aquatic Ecosystem Research Lunz am See Austria; ^7^ Norwegian Institute for Nature Research Oslo Norway

**Keywords:** biodiversity, Community Ecology, fish, freshwater lakes, phytoplankton, zooplankton

## Abstract

Covariation in species richness and community structure across taxonomical groups (cross‐taxon congruence) has practical consequences for the identification of biodiversity surrogates and proxies, as well as theoretical ramifications for understanding the mechanisms maintaining and sustaining biodiversity. We found there to exist a high cross‐taxon congruence between phytoplankton, zooplankton, and fish in 73 large Scandinavian lakes across a 750 km longitudinal transect. The fraction of the total diversity variation explained by local environment alone was small for all trophic levels while a substantial fraction could be explained by spatial gradient variables. Almost half of the explained variation could not be resolved between local and spatial factors, possibly due to confounding issues between longitude and landscape productivity. There is strong consensus that the longitudinal gradient found in the regional fish community results from postglacial dispersal limitations, while there is much less evidence for the species richness and community structure gradients at lower trophic levels being directly affected by dispersal limitation over the same time scale. We found strong support for bidirectional interactions between fish and zooplankton species richness, while corresponding interactions between phytoplankton and zooplankton richness were much weaker. Both the weakening of the linkage at lower trophic levels and the bidirectional nature of the interaction indicates that the underlying mechanism must be qualitatively different from a trophic cascade.

## Graphical abstract

Species richness is known to be affected by spatial dispersal, local environmental filtering, and biotic interactions. After adjusting for known dispersal gradients (longitude) and local filtering (total phosphorus (TP) and organic carbon (TOC)), we find positive, reciprocal interactions between species richness at three trophic levels (phytoplankton, zooplankton, and fish) across 73 lakes in Southern Norway and Sweden. While we found strong congruence across trophic levels in species richness and community structure, we did not find support for unidirectional top‐down or bottom‐up effects, as would have been expected from a trophic cascade.

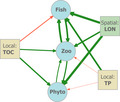

## 1 INTRODUCTION

Positive covariation in diversity and community structure across taxonomical groups, which we in accordance with Bilton, Mcabendroth, Bedford, and Ramsay (2006) will call cross‐taxon congruence, can result from biotic interactions such as predation or parasitism, or it can be merely a consequence of different organismal groups responding similarly to environmental gradients or biogeographical dispersal factors (e.g., Rooney & Azeria, 2015). Several studies have found cross‐taxon congruence to be weak, which in turn implies limitations on our ability to predict the diversity of one taxonomical group when given information about another (Gaston, 2000; Gioria, Bacaro, & Feehan, 2011; Heino, 2010). However, there are still only a limited number of reported biodiversity studies across multiple trophic levels (e.g., Qian & Ricklefs, 2008; Sandom et al., 2013; Zhang et al., 2018). This is especially the case for aquatic habitats (but see Collen et al., 2014). Indeed, the literature on cross‐taxon congruence often does not clearly distinguish between studies within and across trophic levels. For example, 10 of the 24 aquatic case studies reviewed by Heino (2010) are on the same trophic level (e.g., zooplankton or macroinvertebrate groups) or in different habitats (e.g., littoral macrophytes and profundal zoobenthos).

### 1.1 Causes and consequences of congruence

Biological indicators are increasingly used for monitoring environmental change, such as in the European Union Water Framework Directive (Allan et al., 2006). If there is, in fact, only limited cross‐taxon biodiversity congruence, this could have practical consequences for the utility and functionality of indicator species or surrogate taxa in conservation biology (Santi et al., 2010). For example, a recent meta‐analysis (Westgate, Tulloch, Barton, Pierson, & Lindenmayer, 2017) concluded that no single taxon was an optimal surrogate for both the richness and composition of unmeasured taxa unless adjusted for covariates like spatial extent, grain size, and latitude.

Cross‐taxon congruence also has theoretical ramifications for understanding the mechanisms that maintain biodiversity and functioning of ecosystems. Classical studies like Brooks and Dodson (1965) and Paine (1966) demonstrate strong cascading effects of top predator abundance on lower trophic levels. These studies focus on top predator biomass (or presence/absence), while fewer studies have tried to investigate whether predator diversity can have similar effects, and most of these have focused on predator diversity effects on biomass rather than diversity at lower trophic levels (e.g., Bruno & Cardinale, 2008; Griffin, Byrnes, & Cardinale, 2013). It is generally accepted that both competition and predation play a role in structuring food webs, and that their relative importance may vary with trophic level (Chesson & Kuang, 2008). Theoretically, increased predator diversity could lead to a more diversified predation pressure that would allow more species to coexist at the prey level which again could cascade to the next trophic level, although the theoretical and empirical support for this type of cascading effect of predator diversity is still inconclusive (Chesson, 2000; Chesson & Kuang, 2008; Levin & Segel, 1982; Nilsson, 2008; Wesner, 2012). In a recent meta‐analysis of food web networks, Turney and Buddle (2016)conclude that species richness generally decreases with trophic level, and that both biotic interactions and environmental constraints play a role in creating this pattern.

While bottom‐up approaches dominate contemporary biodiversity research (e.g., Castagneyrol & Jactel, 2012), Terborgh (2015) points out the need to take top‐down mechanisms into account when explaining the ecological regulation of biodiversity. And, given that trophic cascades are often stronger in lakes than in other habitats (Shurin et al., 2002), one would also expect a higher potential for detecting top‐down effects on biodiversity in lake ecosystems. Several studies have pointed out the importance of spatial scale or grain‐size for cross‐taxon congruence (Qian & Kissling, 2010; Wolters, Bengtsson, & Zaitsev, 2006; Westgate et al., 2017). Compared to other habitats, lake ecosystems have the advantage of well‐defined boundaries that define a single natural scale for studying their biodiversity, especially if the size range of investigated lakes is constrained (cf. Dodson, 1992). While biomass responses in trophic cascades are known to have alternating signs between successive trophic levels (e.g., Jeppesen et al., 2003), recent global analyses from terrestrial environments (e.g., Qian & Ricklefs, 2008; Sandom et al., 2013) indicate that inter‐trophic interaction effects on diversity can be uniformly positive. Similar patterns have also been found in previous studies on fish and zooplankton congruence in Southern Norway (Hessen, Faafeng, Smith, Bakkestuen, & Walseng, 2006; Hobæk, Manca, & Andersen, 2002).

### 1.2 Postglacial dispersal

The Pleistocene glaciation and postglacial recolonization history is a very important, but often neglected, driver of biodiversity and community structure in temperate lakes (Hortal et al., 2014). In Scandinavia, the main recolonization routes of freshwater fish are known to be either from the North Sea in the west (for anadromous species) or through the freshwater stages of the Baltic Sea (Björck, 1995) in the east (Huitfeldt‐Kaas, 1918). While molecular evidence identifies several possible glacial refugia for the eastern recolonization (Kontula & Väinölä, 2001; Nesbo, Fossheim, Vollestad, & Jakobsen, 1999), the end result is a distinct longitudinal gradient in freshwater fish community composition from west to east in Southern Scandinavia. The lakes in this region should thus offer a unique chance to evaluate the relative effects of local biotic interactions versus regional biogeographical processes among three distinctively different taxonomical groups; fish, zooplankton, and phytoplankton.

### 1.3 Design and analysis

Previous analyses of the longitudinal diversity gradient across Norway (Hessen, Bakkestuen, & Walseng, 2007; Hessen et al., 2006) and the Nordic countries (Ptacnik, Andersen, Brettum, Lepistö, & Willén, 2010; Ptacnik et al., 2008) were based on data that were heterogeneous across years, seasons, and lake sizes, and that also had limited information on lake productivity and water chemistry. Previous studies confined to the Norwegian boundaries also had rather narrow longitudinal gradients. In the current study, we expanded this historical research and made a comprehensive, synoptic survey of species diversity of phytoplankton, zooplankton, and fish in lakes along a 750 km east‐west gradient from the North Sea to the west to the Baltic Sea to the east and contrasted this gradient with local covariates for lake productivity (total phosphorus [TP] and total organic carbon [TOC]). Since lake surface area also may impact species diversity (Dodson, 1992), we excluded lakes with surface area <1 km^2^ and depth <4 m. The spatial extent was deliberately chosen to cover a long gradient in biodiversity while minimizing the impacts of climatic factors and human activities.

We first analyze the pair‐wise relationships between species richness and community structure across trophic levels (phytoplankton, zooplankton, and fish). Then, we quantify spatial and environmental sources of variation in species richness and community structure within each trophic level. The variation due to biotic interactions cannot be measured directly but we expect it to be constrained by the variation that is not explained by spatial gradients or environmental conditions. Finally, we analyze all three trophic levels together, which allows us to quantify between‐level interactions while controlling for sources of variation related to dispersal limitation and filtering by the local environment. At this stage, we can also address directionality of the interaction between trophic levels: whether it flows upward from the resource level (bottom‐up), downward from top predator level (top‐down), or bi‐directionally between adjacent levels.

## 2 MATERIAL AND METHODS

### 2.1 Survey design

**FIGURE 1:**
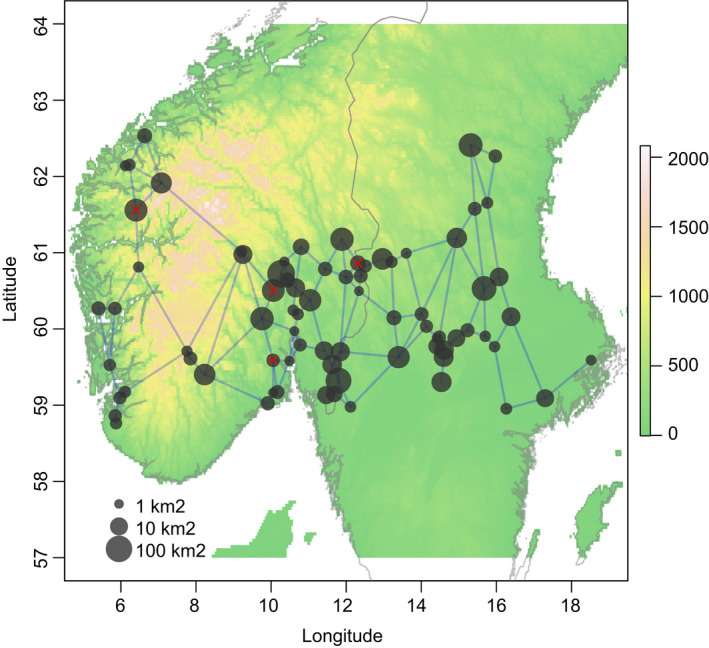
Map showing the locations of the investigated lakes with marker size scaled by the logarithm of lake surface area. Blue lines represent the Gabriel neighborhood (Bivand, Pebesma, & Gómez‐Rubio, 2013) network structure used in the spatial autoregression analyses. The underlying raster shows altitude taken from the WorldClim data base (Hijmans, Cameron, Parra, Jones, & Jarvis, 2005). Notice the central Norway mountain ridge massive extending S‐N around 8°E. Four lakes indicated by red “X” marks were not included in the analysis, either due to missing zooplankton data, glacial influence, or high/low pH

Lakes for this survey were selected using existing data on Norwegian and Swedish lakes from the “Rebecca” (Lyche Solheim et al., 2008) and “Nordic lake survey 1995” (Henriksen et al., 1998) data sets. To constrain the climatic range and the influences of anthropogenic disturbances like acidification and eutrophication, we selected a subset of lakes with latitude 58–63°N, <600 m above sea level, area >1 km^2^, pH > 5, TP < 30 µg/L, and TOC < 30 mg/L. The lakes were chosen to create a representative subset of boreal lakes with uniform spatial distribution and orthogonal gradients of TP, TOC, and longitudinal position. TP and TOC represent two major effects on aquatic productivity (Thrane, Hessen, & Andersen, 2014), while longitude reflects a regional phytoplankton diversity gradient (Ptacnik et al., 2010). Lakes were selected by a stratified random sampling where three gradient variables were split in two factor levels (high/low), giving eight different combinations (strata) of TP, TOC, and longitude. From the 741 lakes in the data base that fit the constraints, a total of 12 lakes were randomly chosen from each of the 8 strata. Sampling was performed mainly by an amphibious aircraft (Cessna 206) in July to August 2011 (Thrane et al., 2014). Due to unfavorable weather conditions during sampling, the number of sampled lakes was eventually reduced to 77 (Figure 1; see Appendix S1 for further details).

### 2.2 Sampling

Five‐liter surface samples (0–5 m depth) were collected with an integrating water sampler (Hydro‐BIOS, Germany) in the central part of each lake during daytime. Concentrations of TP and TOC were measured at two independent laboratories (Norwegian institute for water research [NIVA] and University of Oslo [UiO]). TP was measured on an auto‐analyzer as phosphate after wet oxidation with peroxodisulfate in both laboratories (detection limit 1 µg P/L). TOC was measured by infrared CO_2_ detection after catalytic high‐temperature combustion (Shimadzu TOC‐VWP analyzer (UiO) or Phoenix 8000 TOC‐TC analyzer (NIVA)), with both laboratories reporting detection limits of 0.1 mg C/L. Specific conductivity (COND), as a measure of total dissolved solids was measured in situ with an RBR XRX‐622 profiling multi‐parameter CTD, as well as in the NIVA laboratory. Correspondence between the two laboratories was generally high, such that we used the averaged results in the following analysis.

### 2.3 Phytoplankton

Phytoplankton were analyzed with inverted microscopy on 0–5 m integrated samples fixed with Lugol's solution using the classical Utermöhl method (Lund, Kipling, & Le Cren, 1958) with the same taxonomical nomenclature as in Järvinen et al. (2013). Counting units were generally resolved to species level except for small, naked flagellates which are impossible to identify by light microscopy. The number of counting units varied from 394 to 2,106 per sample, with a median of 784 units. Counts were converted to bio‐volumes by multiplying cell abundances with average cell volumes estimated from measured linear dimensions and geometrical formulae. Each sample was examined at different magnifications to also cover large‐celled species with low abundance, but high contribution to bio‐volume. For rarefaction and diversity estimation, counting unit abundances at different magnifications were normalized to the sample size of the lowest magnification (see Appendix S2 for more details).

### 2.4 Zooplankton

Vertical net hauls were taken from just above the bottom to the surface using a standard zooplankton net with 90 µm mesh size and a diameter of 40 cm. Samples were collected in brown glass bottles, preserved with ethanol, and kept cool and dark until analysis. All crustaceans except cyclopoid and calanoid nauplii were identified to species, using the same taxonomy as in Walseng, Hessen, Halvorsen, and Schartau (2006). A 0.2% to 20% subsample, containing on the average 200 individuals (range 89–544), was counted to record the relative distribution of “common” species. Even though these counts are only available for crustaceans, we will for simplicity refer to them as “zooplankton” in the remainder of the text. Rotifers were not included in this survey, but previous studies from the same region (Hobæk et al., 2002) found practically identical community composition gradients from zooplankton crustaceans alone and from a data set including crustaceans and well as those rotifer species that could be easily identified from preserved samples.

### 2.5 Fish

Fish community data were not observed directly in the survey. We compiled a fish community data set for investigated lakes based on the Norwegian and Swedish biodiversity data information facilities (http://www.artsdatabanken.no/ and http://www.artdatabanken.se/), complemented by telephone or email interviews with local stakeholders as well as national angling associations (http://www.meite.org/, http://alltfiske.se/). Fish data are for obvious reasons restricted to presence/ absence.

### 2.6 Final subset

One glacially influenced lake (temperature < 10°C; Jølstravatnet, Norway) and two lakes with low/high pH (<6 and >8; Halsjøen and Bergsvannet, respectively—both Norway) were omitted from the final data set to reduce the effects of these potential outliers. In one lake (Sperillen, Norway), the sampling had to be aborted due to weather conditions before the zooplankton sample was taken. Accordingly, we ended up using data on environmental gradients and phytoplankton, zooplankton, and fish community structure from 73 lakes in our analysis.

Clearly neither fish nor zooplankton, or even phytoplankton represent homogeneous trophic levels. Most metazoans are omnivores, and many have ontogenetic and seasonal dietary shifts. While there is no way to practically implement this in the analysis, we would still argue that these three taxonomical groups represent trophic levels on a very aggregate level (i.e., no phytoplankton eat zooplankton, no zooplankton eat fish and no fish eat phytoplankton—at least in the communities represented in our study).

### 2.7 Statistical analysis

#### 2.7.1 Explanatory variables

We use latitude, longitude, and altitude (LAT, LON, ALT) as spatial gradient indicator variables, while TP, TOC, and COND, all log transformed, serve as indicators for the local environment. The covariates can also be grouped according to whether they were part of the original gradient design (longitude, TP, and TOC), or represented variables that were incompletely constrained by the study design (latitude, altitude, and COND). Conductivity (COND) is strongly related to pH and total nitrogen, while altitude and latitude both have effects on mean July air temperature, which again is correlated with surface water temperature (see Appendix S1 for further details). Pearson correlations between the gradient variables are generally <0.5, with notable exceptions for longitude and log(TOC) (*r* = .65), and log(TP) and log(TOC) (*r* = .57). The confounding between longitude and TOC gradients is at least partly an unfortunate consequence of having to abandon the last 19 northernmost lakes in the original survey design which were sites with relatively high TP and low TOC. Despite these shortcomings, variance inflation factors (VIFs) in our regression models were generally ≤3, indicating no severe effects of multi‐collinearity (Zuur, Ieno, & Elphick, 2010). Longitude is also strongly correlated with the regional productivity indicator (R‐TP) of Ptacnik et al. (2010), estimated from distance‐weighted interpolation of local TP from an independent data set. Pearson correlation coefficients ranged from 0.93 to 0.97 between longitude and R‐TP with distance thresholds from 100 to 500 km (figure S1 in Khomich, Kauserud, Logares, Rasconi, and Andersen, 2017). R‐TP was also strongly correlated with TOC (*r* = .70–.75), but not with COND or TP (*r* ≤ .11 and *r* ≤ .22, respectively).

#### 2.7.2 Response variables

For phytoplankton and zooplankton, where we had both abundance and counting effort information, we could compute several different measures of diversity, including extrapolation‐ and rarefaction‐based species richness, and also taxon richness at the genus level (G) sensu Ptacnik et al. (2008). We also computed Shannon (*H*) and Simpson (*D*) diversity indices as the first and second order Hill numbers (exp(*H*) and 1/*D*). All the richness indicators, and to some extent the information theory‐based diversity indices, were strongly correlated with the observed species richness (see Appendix S2 for details). We thus chose to base all subsequent diversity analyses on observed species richness. We estimated beta diversity, or the number of species turnovers along the complex‐gradients of a landscape, by the ratio of total richness (gamma diversity) to the average local richness (alpha diversity) as recommended by (Tuomisto, 2010).

Community structure data were summarized by nonmetric multidimensional scaling (NMDS) ordination from multiple starting points (metaMDS function in the vegan package [Oksanen et al., 2019]), using Bray‐Curtis or Jaccard dissimilarities as distance measures. We used variance partitioning by redundancy analysis (RDA, using varpart function in vegan) on Hellinger transformed data (Borcard, Legendre, & Drapeau, 1992) to disentangle spatial community gradients (LAT, LON, and ALT) from the effects of the local environment (TOC, TP, and COND). Based on the simulation analysis of (Gilbert & Bennett, 2010), we represented the spatial gradients as an up to the second order response surface in latitude and longitude.

We partitioned the variance components explained by different groups of covariates, sensu Borcard et al. (1992), by fitting linear models using groups of explanatory variables alone or together and solving a system of linear equations. As indicated by the simulation results of Gilbert and Bennett (2010) and the meta‐study of Soininen (2014), using a spatial representation based on polynomial surfaces is expected to give a more conservative spatial variance contribution than methods based on eigenvector representations.

Within the constraints of the study design, we have no direct measure of biotic interactions per se. But we expect that variation due to biotic interactions to be at least partly contained in the variance that is not explained by the spatial and environmental covariates. We can estimate this variance contribution by using species richness at adjacent trophic levels as co‐variates.

#### 2.7.3 Spatial analysis

We used the Moran's *I* statistic to test for spatial autocorrelation in regression model residuals. Autocorrelation tests were based on Gabriel neighborhoods with spatial weights calculated from a triangulation network of Euclidean inter‐lake distances (Figure 1). Permutation‐based tests (function moran.mc from the spdep package for R; (Bivand & Piras, 2015)) used the default row standardization of spatial weights. Mantel permutation tests (function mantel in the vegan library) were used to investigate relationships between community dissimilarities and geographical distances (computed by the spDists function from the spdep library).

Community similarity distance decay scales (Soininen, McDonald, & Hillebrand, 2007) were estimated by fitting decaying exponential functions to similarity—distance relationships (Nekola & White, 1999). Community similarities were computed as 1‐ Jaccard or Bray‐Curtis dissimilarity. Since the n(n-1)/2 similarities between *n* communities would not be independent, we used the average similarities in 40 equiprobable distance classes as input to the regression models (Shurin, Cottenie, & Hillebrand, 2009). The half‐distance scale of similarity distance decay (Soininen et al., 2007) was calculated as -log(2)/α, where *α* is the rate constant of the fitted exponential decay relationship.

### 2.8 Structural and simultaneous equation models

Multivariate relationships between study design covariates and species richness at all three trophic levels were explored by two unrelated approaches: nonrecursive (bidirectional) simultaneous equations models following (Zhang et al., 2018) and recursive (unidirectional) structural equation models (Grace, 2006; Pearl, 2009), using the systemfit (Henningsen & Hamann, 2007) and lavaan (Rosseel, 2012) packages, respectively. Specifically, we use the original study design variables (latitude, TP, and TOC) as indicators of dispersal and local environment, and species richness at adjacent trophic levels as indicators of biotic interaction. The unidirectional structural equation models allow us to investigate models with contrasting causality (top‐down vs. bottom‐up), while the bidirectional simultaneous equation models do not. All variables used in structural/ simultaneous equation model fits were standardized to zero mean and unit standard deviation to simplify comparison of coefficient estimates and covariances.

All statistical analyses were performed in the R 3.5.3 statistical computing environment (R Core Team, 2019).

## 3 RESULTS

### 3.1 Phytoplankton diversity and community composition

A total of 395 taxa were identified in the phytoplankton samples, of which 106 were found in just one lake and 187 taxa occurred in less than 4 lakes. Observed phytoplankton taxon richness varied fourfold from 23 to 105 with a mean and median of 58 taxa/ lake. The corresponding beta diversity (395/58 = 6.8) indicates more than sixfold phytoplankton taxon inventory turnover across the gradient. Of the 395 identified taxa, 356 belonged to the 10 classes *Synurophyceae*, *Cryptophyceae*, *Euglenophyceae*, *Trebouxiophyceae*, *Dinophyceae*, *Bacillariophyceae*, *Chrysophyceae*, *Cyanophyceae*, *Zygnematophyceae*, and *Chlorophyceae* (12, 15, 16, 23, 25, 42, 50, 54, 59, and 60 taxa, respectively). Based on the general similarity between phytoplankton ordinations for different subsets and methods (see Appendix S3 for details), we chose to base the phytoplankton community analysis on relative biovolume fractions of the 208 taxa represented in >3 lakes. It should be noted that this subset has a beta diversity (208/54 = 3.9) which is more like those for zooplankton and fish (beta diversity = 3.1 and 3.9, respectively; see below).

### 3.2 Zooplankton diversity and community composition

Of the 47 crustacean zooplankton species encountered, 34 were considered truly pelagic, while the remaining 13 are known to have a predominantly littoral distribution (Walseng et al., 2006). Since zooplankton species richness estimated from combinations of common/rare pelagic/littoral species were all highly correlated (data not shown), we chose to focus our analysis on the subsample counts of the 34 pelagic species. In this subset, species richness varied from 5 (in two of the most western lakes) to 19, with a mean and median of 11 species per lake. The corresponding beta diversity (34/11 = 3.1) indicates a nominal 3‐fold zooplankton species inventory turnover across the gradient. More than half of the zooplankton species were from families *Cyclopidae*, *Daphniidae*, and *Diaptomidae* (8, 7, and 5 species, respectively), while *Sididae*, *Bosminidae*, *Temoridae* were also represented with more than 1 species.

### 3.3 Fish diversity and community composition

The 31 fish species varied in occupancy (number of lakes where the species occurred) from 2 to 61 of the 73 lakes. Fish species richness varied from 1 to 23, with a mean and a median of 8 species/ lake, and as for phyto‐ and zooplankton, with a distinct increase from west to east. The corresponding beta diversity (31/8 = 3.9) indicates an almost 4‐fold fish species inventory turnover across the gradient. More than half of the fish species were from families *Cyprinidae* and *Salmonidae* (12 and 6 species, respectively), while *Cottidae*, *Percidae*, and *Petromyzontidae* were also represented with more than 1 species.

### 3.4 Diversity and community composition across trophic levels

The explanatory variables formed the same general grouping pattern across all ordinations (see Appendix S3 for details). Longitude and log(TOC) vectors were generally close together and orthogonal to the other four variables. The latitude and altitude vectors were generally in the opposite direction of the log(TP) and log(COND), reflecting that local (TP) and regional (COND) productivity indicators were closely related, and that higher altitude/ latitude lakes appear less productive. It is notable that species richness (as indicated by marker sizes in figure S3.1/3/5) generally increased in the direction of the longitude/ TOC vectors.

**FIGURE 2:**
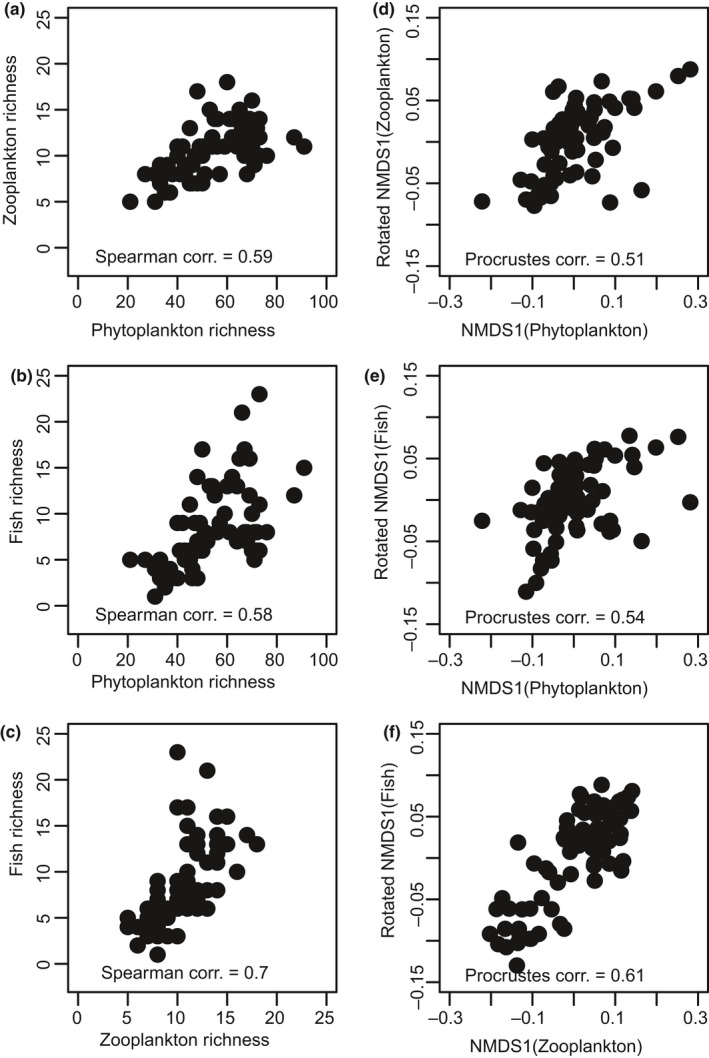
Scatterplots of species richness (left) and first NMDS axis scores (right) between phytoplankton, zooplankton and fish communities, with corresponding Spearman or Procrustes correlation coefficients indicated. NMDS scores on the vertical axes have been Procrustes rotated to facilitate interpretation

Figure 2 summarizes the general congruence between diversity measures and community ordination axis scores across the three trophic levels. Species richness was highly correlated for phytoplankton, zooplankton, and fish with Spearman correlations *ρ* = 0.58–0.70. Procrustes rotated site scores (Peres‐Neto & Jackson, 2001) on the first NMDS axis were also highly correlated, while correlations between second NMDS axis scores were much weaker (*ρ* = 0.24–0.31). Cross‐taxon congruence, as measured by pair‐wise correlations in species richness and community structure, was consistently higher in our study than the average correlation of 0.38 found in a meta‐analysis by Wolters et al. (2006). It is also worth noting that correlations were generally higher between adjacent trophic levels (phytoplankton/zooplankton and zooplankton/fish; *ρ* = 0.54 and 0.67, respectively) than between phytoplankton and fish (*ρ* = 0.39) which are two trophic levels apart.

### 3.5 Spatial and local effects on diversity and community composition

Linear regression models for phytoplankton, zooplankton, and fish richness predicted by spatial gradient (latitude, longitude, and altitude) and local environment (TOC, TP, and COND, all log transformed) variables explained 51%, 62%, and 57% of the total variation, respectively. Longitude was by far the best predictor for species richness across all trophic levels, indicating that phytoplankton, zooplankton, and fish richness increased by 2.33 ± 0.56, 0.44 ± 0.09, and 0.73 ± 0.15 taxa per degree longitude eastward. Models with a second order polynomial term in latitude and longitude were only marginally better than the ones with linear terms for latitude and longitude (likelihood ratio test *p*‐values = .03, .08, and .60 for phytoplankton, zooplankton, and fish models, respectively).

### 3.6 Variance partitioning: species richness

**FIGURE 3:**
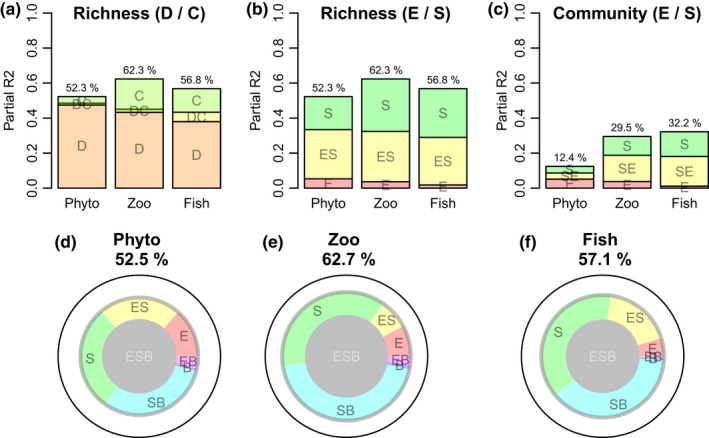
Variance partitioning of phytoplankton, zooplankton, and fish species richness or community structure. (a) Species richness variance partitioned between variables representing study design (D; longitude, total organic C, total P) and constraints (C; latitude, altitude, conductivity). Species richness (b) and community composition (c) variance partitioned contributions from local environment (E; conductivity, total organic C, total P), spatial position (S; latitude, longitude, altitude), or not being resolvable between the two (ES). (d–f) Species richness variance at each trophic level partitioned between local environment (E), spatial position (S), and species richness at adjacent trophic levels (B), as well as unresolvable 2‐ and 3‐way combinations (ES, SB, BE, ESB)

Figure 3a shows that the original study design covariates (longitude, TP, TOC) explained far more of the species richness variance than the constraint covariates (altitude, latitude, and COND). The fraction of species richness variation that could be uniquely explained by local environment was small for all taxon groups (5.3%, 3.7%, and 1.8% for phytoplankton, zooplankton, and fish, respectively; Figure 3b). A substantial fraction could be explained by spatial gradient variables (18.9%, 29.9%, and 27.8%), even with a deliberately conservative spatial representation. Almost half of the explained variation could not be resolved between local and spatial factors (28.1%, 28.7%, and 27.2% of total variation), most likely due to unavoidable confounding between longitude and TOC, and between altitude/latitude and TP/COND. Moran's *I* tests indicated that the full regression models for species richness had only weak spatial autocorrelation in their residuals (*p*‐values based on 999 permutations = 0.091, 0.516, and 0.306 for phytoplankton, zooplankton, and fish, respectively). From this, we conclude that the linear effects of latitude, longitude, and altitude captured the majority of the spatial variation in species richness.

### 3.7 Variance partitioning: community composition

Communities vary in many more properties than those captured by univariate diversity measures, although the total community variability should, to some extent, scale with the gamma diversity of a given organism group. Direct gradient analyses by RDA with Hellinger transformed community data and the same indicator variables for local (E) and spatial (S) gradients as above seem to reflect such a pattern (Figure 3c): Only 12.4% of the total variance could be explained for phytoplankton, the most species‐rich trophic level, while 29.5% and 32.2% could be explained for zooplankton and fish which have substantially lower and quite similar species richness. Partitioning community variances by the method of Borcard et al. (1992) indicated relatively higher importance of the local environment for phytoplankton (5.1% of total variance) than for the higher trophic levels (3.8% and 1.1% for zooplankton and fish, respectively). In contrast, the variance fraction uniquely explained by spatial gradients increased with trophic level: 3.8%, 10.8%, and 14.1%. As with the regression models for species richness, there was a substantial part of the explained variance that could not be resolved between spatial and local environmental gradients: 3.5%, 15.0%, and 17.0% for phytoplankton, zooplankton, and fish, respectively. 3‐way variance partitioning using species richness at adjacent trophic levels as biotic (B) covariates (Figure 3d‐f) revealed very small variance contributions from biotic interactions alone, but with possible biotic contributions that could not be resolved from spatial (SB) or spatial/ environmental effects (ESB).

### 3.8 Distance decay of community similarity

Community dissimilarities increased with geographical distance across all trophic levels (Mantel correlations = 0.36, 0.45, and 0.64 for phytoplankton, zooplankton, and fish, respectively, all with *p* = .001 on 999 permutations). Similarity decay half‐distances, at which similarity has decayed to half of the similarity at zero distance (Soininen et al., 2007), were estimated to 1,237, 294, and 317 km for phytoplankton, zooplankton, and fish, respectively. Approximate 95% confidence limits were overlapping for zooplankton and fish community similarity decay half‐distances (260–337 km and 289–350 km), which were both nonoverlapping with that of phytoplankton (1,085–1,434 km).

### 3.9 Simultaneous and structural equation models of trophic interaction

**FIGURE 4:**
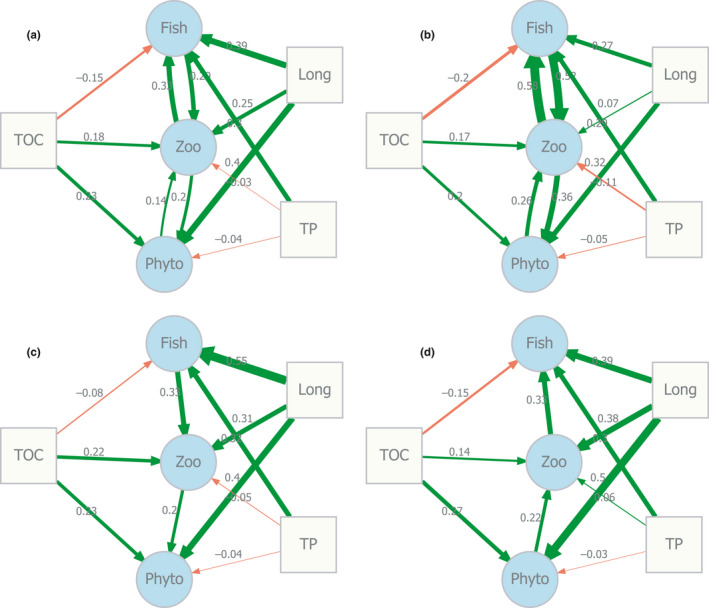
Network representations of simultaneous and structural equation models relating species richness at adjacent trophic levels to study design covariates (longitude, total organic C, total P). The simultaneous equation model in panel a is fitted with ordinary least squares (OLS) assuming independent error terms in the three simultaneous model equations, while panel b is fitted with generalized least squares (“Seemingly Unrelated Regressions”—SUR) which can represent covariances between model equation error terms. The structural equation model networks in panels c and d represent unidirectional top‐down (C) and bottom‐up (D) interactions between trophic levels

Due to identifiability constraints in the structural equation model, we used only the 3 original study design variables (longitude, total P, TOC), which explained most of the species richness variance (Figure 3a). The bidirectional models (Figure 4a,b) explained 60% and 84% of the total variance (McElroy's *R* ^2^), depending on whether errors were assumed uncorrelated (A) or correlated between equations (B). The unidirectional models (Figure 4c,d) had equation‐wise *R* ^2^ ranging from to .43 to .49 for both the top‐down (C) and bottom‐up (D) models. The path coefficients are consistent with strong bidirectional interactions between zooplankton and fish, but less so between zooplankton and phytoplankton, and thus not indicative of a unidirectional trophic cascade across all trophic levels.

## 4 DISCUSSION

We found a striking congruence among phytoplankton, zooplankton, and fish communities in large lakes across Southern Scandinavia. The small independent contribution from the local environment compared with spatial factors (Figure 3) indicates a minor role for classical species sorting or environmental filtering in the plankton community assembly of these lakes. If this is the case, then the potential for using plankton composition as a predictor for the local environment is probably less than expected, at least for low‐productivity lakes like the ones investigated here. This leads to the paradoxical situation that due to strong congruence there is an unexpectedly high potential for using diversity at one trophic level as proxy or surrogate for the whole community, but this does not mean that this surrogate will be a good indicator for the local environment. The study design constrained most of the spatial variation to a 750 km longitudinal transect, from the North Sea in the west to the Baltic Sea in the east. The freshwater fish species distribution along this gradient is thought to reflect species‐specific differences in postglacial recolonization depth over the last 10,000 years, which to a large extent can be explained mechanistically in terms of hydrological connectivity and migration barriers (Spens, Englund, & Lundqvist, 2007). Although human‐mediated dispersal is also relevant for some species, historical dispersal limitation is considered the major determinant for the present distribution pattern of fish in Scandinavian lakes.

### 4.1 Migration barriers

Zooplankton can bypass hydrological migration barriers by, for example, phoretic dispersal with animal vectors like birds and flying stages of insects (Bilton, Freeland, & Okamura, 2001). Although there is no consensus on the actual rates of zooplankton dispersal (Bohonak & Jenkins, 2003), it is likely that passively dispersing invertebrates would penetrate further than fish on the millennial time scale of postglacial colonization. In our study, this expectation is challenged by the overlapping confidence intervals for fish and zooplankton community similarity decay half‐distances. Large‐scale studies from North America also suggest modest migration constraints, and minor contribution from local filtering, but that climate and colonization constraints related to Allee effects could be major structuring forces (Henriques‐Silva, Lindo, & Peres‐Neto, 2013; Pinel‐Alloul et al., 2013). We found no indication of Allee effects in the sense that the parthenogenetic cladocerans were not more successful than the sexually reproducing copepods in the sites with lowest species richness.

Phytoplankton had weaker similarity distance decay scaling than zooplankton and fish, as indicated by a fourfold longer half‐distance. This pattern is consistent with other lentic studies reviewed by (Jenkins, 2014) (table S2 therein), where phytoplankton appear to have weaker distance decay than metazoan communities. This pattern is consistent with a general, inverse relationship between spatial turnover and organism size in freshwater communities, as reported by (Shurin et al., 2009). Higher dispersal capacity in phytoplankton is one possible explanation, but spatial structure need not reflect dispersal limitation since longitude is also strongly aliased with regionally averaged TP (R‐TP; Ptacnik et al., 2010) in this region. The longitudinal phytoplankton diversity gradient can thus also be explained by meta‐community dynamics in combination with landscape productivity (sensu Ptacnik et al., 2010). The fivefold higher richness of phytoplankton (median = 58 taxa/lake) than higher trophic levels (median = 11 and 8 taxa/lake for zooplankton and fish, respectively) could also indicate that different structuring processes are involved at the base of the food web than at higher trophic levels.

### 4.2 Top‐down effects

Apex predator abundance has been shown to promote higher diversity at lower trophic levels by suppressing otherwise dominating species (Terborgh, 2015), while the role of diversity per se at the predator level is less explored (but see Matias et al., 2017). The dramatic size‐selective effects of visual predators on freshwater zooplankton communities have been known for at least 50 years (Brooks & Dodson, 1965), although much of the classical research has been focused on productivity and trophodynamics rather than species richness. Still there are clear indications that fish richness promotes zooplankton diversity (Hessen et al., 2006; Hobæk et al., 2002). While it is tempting to explain the strong concordance across three trophic levels by unidirectional top‐down processes, closer examination (Figure 4) indicates that the interactions are bidirectional and also becoming weaker with decreasing trophic level.

### 4.3 Representativity

The use of stratified random sampling entails that our inferences can be logically extended to other lakes in the region. By choosing a synoptic sampling strategy, we deliberately emphasize the comparability between lakes at the expense of within‐lake representability. Some studies indicate a close correspondence between snapshot studies of biodiversity and long‐term aggregates (Shurin et al., 2007), which is also supported by other studies finding order of magnitude higher between‐lake variance in taxon richness compared to repeated within‐lake sampling (Allen et al., 1999). We have used community composition data based on direct observation for the two lower trophic levels, while fish community data are constructed from historical observations and public data bases. Fish are long‐lived organisms with slow community dynamics compared to phyto‐ and zooplankton, and while there will be extra uncertainty in this type of data due to, among other things, nonuniform sampling time and effort, the risk of missing a fair number of rare species is minimal compared with that of plankton where net‐hauls necessarily comprise a tiny fraction of the lake volume. Quality of public fish distribution data bases has also been shown to be sufficient for revealing interesting macro‐ecological patterns (Gardezi & Gonzalez, 2008; Griffiths, 2006). It is notable that Griffiths (2006) (figure 6b therein) reported practically the same relationship between longitude and fish species richness as in our study, but over a wider geographical range and from an independent data source (Limnofauna Europaea).

### 4.4 Comparison with other studies

Our study of 73 lakes over a 750 km transect has an extent comparable to many lake biodiversity studies. For example, the median study covered 100 lakes over 780 km in the lentic studies listed in table S2 of Jenkins (2014). We found that 51%–62% of the variance in richness and 19%–30% in community structure could be explained by spatial and environmental predictors. The corresponding residual variance (38%–49% and 70%–81%, respectively) is either lower or similar to many comparable studies of freshwater biodiversity, such as Declerck et al. (2005), Beisner, Peres‐Neto, Lindström, Barnett, and Longhi (2006), Soininen and Luoto (2012), and Viana et al. (2016). Despite these general similarities, most other studies find much weaker evidence for cross‐taxon congruence in freshwater communities than what we have observed (Allen et al., 1999; Declerck et al., 2005; Heino, 2010; Longmuir, Shurin, & Clasen, 2007; Özkan et al., 2014; Vilmi, Karjalainen, Nokela, Tolonen, & Heino, 2016). This discrepancy could be related to either the properties of the study design or the study area. Constraining the ranges of surface area, climate, and productivity in our study design probably increased the effect size of cross‐taxon congruence among other sources of variation. The main difference, though, is probably the strong fish community composition gradient covered in our study, which is largely a consequence of Scandinavian topography over the postglacial re‐colonization time frame. In contrast to zooplankton and especially phytoplankton, there is strong consensus that the Scandinavian fish community gradient is caused by dispersal limitation (Hein, Öhlund, & Englund, 2011; Huitfeldt‐Kaas, 1918; Nilsson & Pejler, 1973; Spens et al., 2007).

## 5 CONCLUSIONS

The strong signal of spatial gradients (S) and their interaction with environment (ES) suggest that landscape influences are more important for driving species richness than biotic interactions and environmental filtering in this region. We see consistent species richness and community composition congruence across trophic levels, but without strong evidence for a single over‐arching mechanism like a top‐down biodiversity cascade (Terborgh, 2015). The bidirectional nature of the biotic interactions, especially at the higher trophic levels, is consistent with the findings of Zhang et al. (2018) for terrestrial plants and consumers. The decreasing strength of these interactions toward the bottom of the food web could indicate higher importance of other factors like landscape productivity and connectivity in shaping phytoplankton community structure (Ptacnik et al., 2010), than for higher trophic levels.

## CONFLICT OF INTEREST

The authors declare no conflict of interest.

## AUTHOR CONTRIBUTION

**Tom Andersen:** Conceptualization (equal); Data curation (lead); Formal analysis (lead); Funding acquisition (equal); Investigation (equal); Methodology (equal); Project administration (lead); Resources (supporting); Visualization (lead); Writing‐original draft (lead); Writing‐review & editing (lead). **Dag O. Hessen:** Conceptualization (equal); Formal analysis (supporting); Investigation (equal); Methodology (equal); Project administration (supporting); Resources (supporting); Writing‐original draft (supporting); Writing‐review & editing (supporting). **Johnny P. Håll:** Investigation (equal); Resources (supporting). **Maryia Khomich:** Formal analysis (supporting); Investigation (equal); Writing‐original draft (supporting). **Marcia Kyle:** Investigation (equal); Methodology (supporting); Project administration (supporting); Resources (supporting); Writing‐original draft (supporting); Writing‐review & editing (supporting). **Markus Lindholm:** Conceptualization (supporting); Methodology (supporting); Project administration (supporting); Resources (supporting); Writing‐original draft (supporting). **Serena Rasconi:** Conceptualization (supporting); Investigation (equal); Methodology (supporting); Resources (supporting). **Birger Skjelbred:** Data curation (supporting); Investigation (equal); Methodology (supporting); Resources (equal). **Jan‐Erik Thrane:** Conceptualization (supporting); Investigation (equal); Methodology (supporting); Resources (equal). **Bjørn Walseng:** Data curation (supporting); Investigation (equal); Resources (equal).

## Supporting information

Appendix S1

Appendix S2

Appendix S3

## Data Availability

All data and scripts used in the analyses are made available in a Github repository (https://github.com/tomand‐uio/congruence) and stored permanently on DataDryad (https://doi.org/10.5061/dryad.7m0cfxprd).
