# Supplementary material for: Congruence, but no cascade—Pelagic biodiversity across three trophic levels in Nordic lakes
Source: Ecol Evol. 2020 Jul 17;10(15):8153–65. doi: 10.1002/ece3.6514 (PMC7417247; doi:10.1002/ece3.6514)
Supplement: Supplementary file 1 — Appendix S1 [file ECE3-10-8153-s001.pdf]

Supplementary information 1: Selection of covariates

Study design is reflected in choice of explanatory variables

The selected lakes were spread out along an W-E longitudinal transect from Western Norway to Eastern Sweden. Using existing monitoring data (REBECCA data base), they were selected to have lake water concentrations of total organic carbon (TOC) and total phosphorus (TP) as uncorrelated as possible to each other, and also to longitudinal position. The lakes were also constrained by latitude (58°N - 62°N), altitude (< 600m), and surface area (>1 km2), as well as keeping TOC < 30 mg/L, TP < 30 \(\mu\)g/L, and pH > 6. Logitude, TOC, and TP are thus the 3 main design variables of this study. Despite the spatial constraints on latitude and altitude, there was still unavoidable variation along these dimensions. To control for this, we included latitude and altitude among the main covariates. Finally, we included conductivity as a 6th covariate, as an indicator of soil depth and landscape productivity which is less sensitive to local pollution than total phosphorus. The set of available environmental data is assembled from various sources, including maps and other georeferenced data (altitude, position, climate), field measurements from the sampling (Secchi depth, CTD casts), as well as chemical analyses on collected water samples

```
library(car)
library(raster)
library(vegan)
```

Climatic indicators: Mean temperatures from Worldclim

We start with extracting lake coordinates from the file “COMSAT 2011 lakes.txt” and use the lake coordinates to extract mean temperature data for each lake from the worldclim data base http://www.worldclim.org/ using the raster package

```
lake <- read.table("data/COMSAT 2011 lakes.txt", header=TRUE, sep="\t")

WGS84 <- CRS("+proj=longlat +ellps=WGS84 +datum=WGS84 +no_defs")
lake.sp <- SpatialPointsDataFrame(lake[, c("Longitude", "Latitude")], lake, proj4string = WGS84)

tmean <- getData('worldclim', var='tmean', res=2.5)
```

The data are mean monthly temperatures over the period 1960 to 1990. We can for example look at the July temperature raster with sampling locations overlaid:

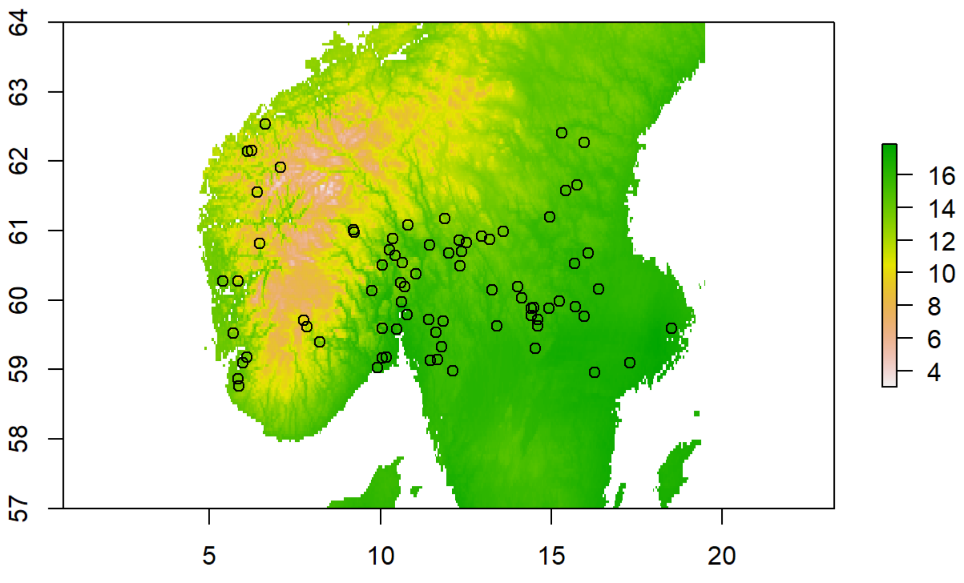

Fig. S1.1 Mean July temperature with sampled lake locations indicated

We then use the raster-function `extract()` to interpolate mean monthly temperatures for each lake. From this we compute annual mean temperature and add this and the mean July temperature (since this was the time of sampling) to the `data.frame` with specific lake information.

```
tmean.lake <- extract(tmean.scand, coordinates(lake.sp))
tmean.lake <- as.data.frame(tmean.lake / 10) # degrees Celsius

lake$Tmean <- rowMeans(tmean.lake)
lake$Tjuly <- tmean.lake[, "tmean7"]
```

We then load some other files containing in situ measurements, including CTD cast averages over 0 - 5 meter (same interval as integrated chemistry samples), as well as some water chemistry measurements:

```
field <- read.table("data/COMSAT 2011 field.data.txt", header=TRUE)
ctd <- read.table("data/COMSAT 2011 CTD.txt", header=TRUE, sep="\t")
chem <- read.table("data/COMSAT 2011 chemistry.txt", header=TRUE)
```

Merge spatial data with other variables

Since all 4 tables have a column `ID` with unique lake identifiers, and no other column names in common we can easily merge the columns we need into one common data frame:

```
env <- lake[, c("ID", "Lake", "Date", "Latitude", "Longitude", "Altitude", "Area.km2", "Tmean", "Tjuly")]
env <- merge(env, field[, c("ID", "Secchi.depth")])
env <- merge(env, ctd[, c("ID", "Temp", "Chla")])
env <- merge(env, chem[, c( "ID", "Cond", "pH", "TOC", "TN", "TP", "SiO2")])
```

We inadvertently ended up including one glacier-influenced lake with temperature < 10C, one acid lake with pH < 6, and two very eutrophic lakes with pH > 10 among our sampling locations. In order to reduce sensitivity to the these extreme lakes, we chose to filter them out:

```
env <- subset(env, (Temp > 10) & (pH > 6) & (pH < 8))
```

The resulting table has 73 observations on 15 spatial, climatic and environmental indicators with the following summey statistics:

Table S1.1. Summary statistics of 15 relevant spatial, climatic, and environmental covariates (1st, 2nd (= median), 3rd quartiles, and maxima)

	Min	Q1	Q2	Q3	Max	Legend
Latitude	58.76	59.59	60.13	60.81	62.53	Latitude
Longitude	5.40	9.91	11.68	14.48	18.52	Longitude
Altitude	7.00	72.00	163.00	260.00	540.00	Altitude (m)
Area.km2	1.09	1.98	3.37	13.36	140.09	Surface area (km2)
Tmean	0.80	3.48	4.64	5.94	7.12	Annual mean temperature (C, Worldclim)
Tjuly	10.50	13.70	15.10	15.90	17.10	Mean July temperature (C, Worldclim)
Secchi.depth	1.05	2.00	2.50	3.90	12.50	Secchi depth (m)
Temp	12.16	16.41	18.02	19.63	21.41	Water temperature (C, CTD)
Chla	4.27	8.55	10.86	12.24	14.80	Chlorophyll (in vivo, CTD)
Cond	0.67	2.33	3.32	5.56	22.60	Specific conductivity (mS/m)
pH	6.29	6.86	7.04	7.21	8.00	pH
TOC	0.34	4.06	6.53	7.62	12.89	Total organic C (mg/L)
TN	0.09	0.22	0.30	0.47	1.53	Total N (mg/L)
TP	1.00	3.00	4.55	7.25	27.45	Total P (?g/L)
SiO2	0.22	1.79	3.02	3.96	5.78	Silicate (mg/L

Some of these variables a noticably right-skewed. We therefore choose to log-transform Secchi depth, surface area, and all the water chemistry variables except pH:

```
env[, c(7, 10, 12:13, 15:18)] <- log(env[, c(7, 10, 12:13, 15:18)])
names(env) <- c("ID", "Lake", "Date", "Latitude", "Longitude", "Altitude", "log.Area",
                "Tmean", "Tjuly", "log.Secchi", "Twater", "log.Chla", "log.Cond",
                "pH", "log.TOC", "log.TN", "log.TP", "log.SiO2")
```

We can consider two groups of main variables: indicators of spatial position (Latitude, Longitude, Altitude), and indicators of local

environmental conditions (log(Cond), log(TOC), log(TP)).

```
main.vars <- c("Latitude", "Longitude", "Altitude", "log.Cond", "log.TOC", "log.TP")
aux.vars <- c("log.Area", "Tmean", "Tjuly", "log.Secchi", "Twater", "log.Chla", "pH", "log.TN", "log.SiO2")
sp.vars <- c("Latitude", "Longitude", "Altitude", "log.Area", "Tmean", "Tjuly", "Twater")
loc.vars <- c("log.Cond", "log.TOC", "log.TP", "log.Secchi", "log.Chla", "pH", "log.TN", "log.SiO2")
```

Relations between main covariates of the study design and other explanatory variables

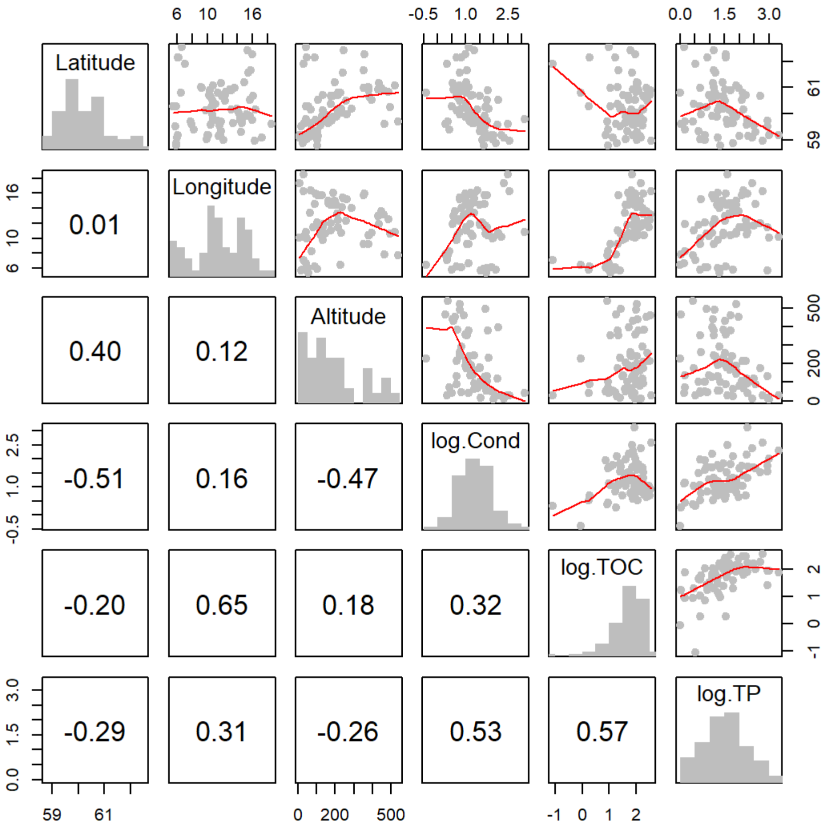

Fig. S1.2 Scatter plot matrix of the 6 main covariates used in this study, with marginal distributions on the diagonal, Pearson correlation coefficient in lower triangle, and LOESS smoothers indicated by red lines

As we can see from the scatter plot matrix above, some of these variables are significantly correlated, especially log(TOC) with longitude (r = 0.65) and log(TP) (r = 0.57). But none of the covariates have variance inflation factors VIF > 3:

Table S1.2. Variance inflation factors for the 6 main covariates

Latitude	Longitude	Altitude	log.Cond	log.TOC	log.TP
1.5	1.8	1.8	1.9	3	2

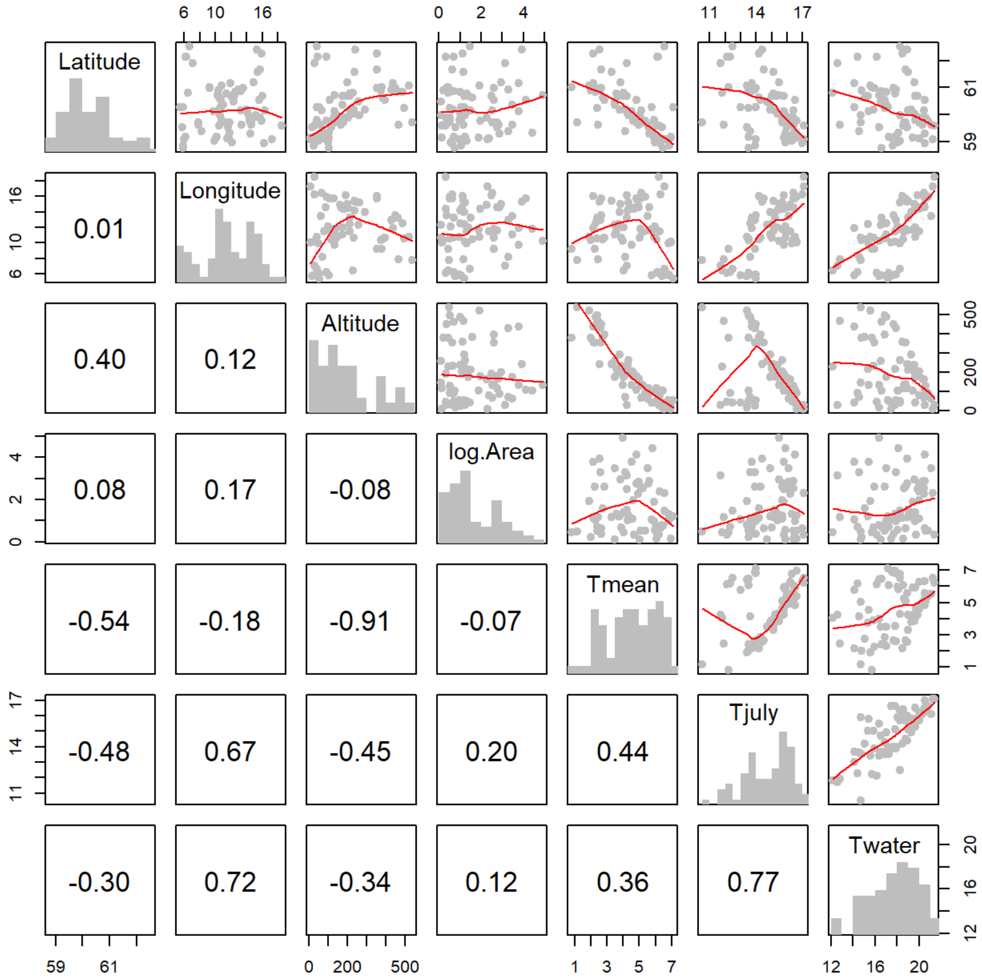

Fig. S1.3 Scatter plot matrix of the 3 spatial indicators together with other geographical and climatic indicators (marginal distributions on the diagonal, Pearson correlation coefficient in lower triangle, and LOESS smoothers indicated by red lines)

Mean annual air temperature is, as expected, negatively related to both Latitude (r = -0.54) and especially Altitude (r = -0.91). Mean July air temperature is more strongly related to Longitude (r = 0.67), reflecting that an West - East transect in this region is also an oceanicity - continentality gradient. Measured water temperature at the sampling is more strongly correlated with mean July air temperature (r = 0.77) than with annual mean temperature (r = 0.36)

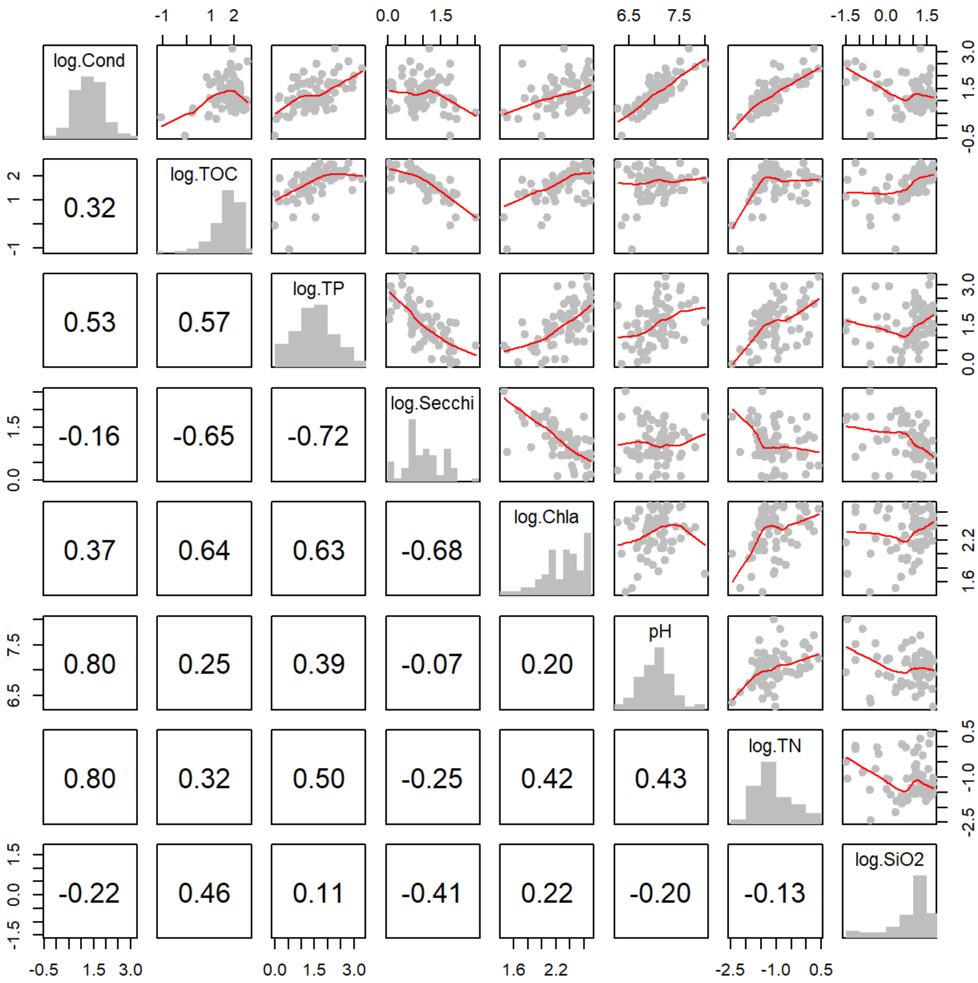

Fig. S1.4 Scatter plot matrix of the 3 local environment indicators together with other water chemistry measurements (marginal distributions on the diagonal, Pearson correlation coefficient in lower triangle, and LOESS smoothers indicated by red lines)

Conductivity (log(Cond)) is strongly positively related to pH and Total N (log(TN); r = 0.80 for both). As such,we can say that log(Cond) also serves as a proxy for pH which was used as a constraint in the lake selection procedure, but which still varied between 6 and 8 in the final data set. Water transparency (log(Secchi)) is, as expected, negatively related to both water color (log(TOC); r = -0.65), algal biomass (log(Chl); r = -0.68), and total P (log(TP); r = -0.72), while chlorophyll and total P are positively related (r = 0.63)

PCA of main and auxiliary covariates

We can illustrate the relationships between the main covariates and other variables as a principal components ordination with passive variables. We first use the rda() function of the vegan package to do an unconstrained ordination of the main variables:

```
env.z <- as.data.frame(scale(env[, -(1:3)]))
rownames(env.z) <-  env$ID

pca <- rda(env.z[, main.vars])
efit <- envfit(pca, env.z[, aux.vars])
```

Table S1.3. Regression coefficients of auxiliary covariates on principal component ordination axes (PC1 and PC2). R2 is the fraction of variance explained, while p is 999 permutation-based significance probability

	PC1	PC2	R2	p
log.Area	0.6388737	-0.7693116	0.0052267	0.822
Tmean	-0.5555771	0.8314650	0.7712902	0.001
Tjuly	-0.9943275	-0.1063615	0.7285961	0.001
log.Secchi	0.8191279	0.5736109	0.4391459	0.001
Twater	-0.9708456	-0.2397059	0.5698506	0.001
log.Chla	-0.9391063	-0.3436267	0.4152432	0.001

	PC1	PC2	R2	p
pH	-0.9904511	0.1378643	0.4092905	0.001
log.TN	-0.8674922	0.4974508	0.6015438	0.001
log.SiO2	-0.0874799	-0.9961663	0.3617439	0.001

In other words, all covariates except log(area) are significantly correlated with the PC ordination axes defined by the 6 main variables:

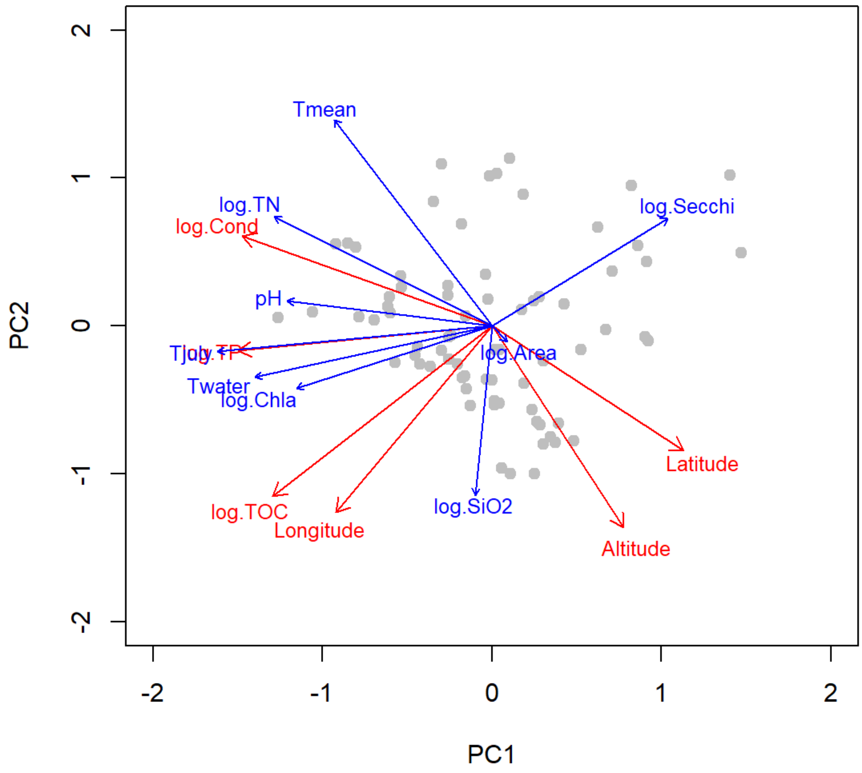

Fig. S1.5 Biplot of a principal component ordination of the 6 main covariates (red arrows), with additional, passively fitted environmental and climatic indicators overlaid as blue arrows and sites as gray dots
