# Supplementary material for: Congruence, but no cascade—Pelagic biodiversity across three trophic levels in Nordic lakes
Source: Ecol Evol. 2020 Jul 17;10(15):8153–65. doi: 10.1002/ece3.6514 (PMC7417247; doi:10.1002/ece3.6514)
Supplement: Supplementary file 2 — Appendix S2 [file ECE3-10-8153-s002.pdf]

Supplementary information 2: Phytoplankton data

Tom Andersen

26 mars 2019

Phytoplankton sampling and analysis

Lugol-fixed phytoplankton samples were taken from integrated 0-5 meter samples from each lake. All samples were analysed by Birger Skjelbred, NIVA, using Utermöhl's sedimentation method and inverted microscopy NS-EN 15204:2006. Count and biovolume data are provided in "long format" with each row representing counts and biovolume of a particular taxon in a particular sample. The text file is converted from the Excel report generated by NIVA's phytoplankton counting data base, but otherwise unaltered

```
env <- read.table("CON env.txt", header=TRUE)
pp <- read.table("data/COMSAT 2011 phytoplankton.txt", header = TRUE, sep = "\t")

pp <- pp[, 1:16]
names(pp) <- c("Lake", "ID", "Date", "Rubin.code", "RebeccaID",
               "Kingdom", "Phylum", "Class", "Order", "Family", "Genus", "Species",
               "Number", "Factor", "Spec.vol", "Bio.vol")

pp <- subset(pp, ID %in% env$ID)

dim(pp)
```

```
## [1] 4268   16
```

The subset of 73 lakes included in the `env` table has 4268 records of 18 variables. The first 3 columns define the sample by lake name, lake ID, and sample date (`Lake` , `ID` , `Date`). The next 2 columns define the taxon as unique 4 + 4 letter genus/species abbreviation codes (`Rubin.code`) and numerical identifiers (`RebeccaID`). The following 7 columns define the taxonomy (`Kingdom` , `Phylum` , `Class` , `Order` , `Family` , `Genus` , `Species`), while the last 5 columns represents the microscopy counts and cell volume measurements: `Number` is the actual number of units counted in the analysed area of the counting chamber. The actual count multiplied by `Factor` gives the number of units per liter of sample. The measure cell volume of the counting unit (μm ^{3}) is given by `Spec.vol` . Biovolume per unit sample volume (`Bio.vol` as mm^3 / m^3 = μg ww / L) is given by the product of `Number` , `Factor` , and `Spec.vol`

Converting biovolume units to detection units

The `Number` field in the `pp` table is in biovolume units, not detection units. This means that a single colony of, say, 8 cells is counted as 8 biovolume units instead of as 1 detection unit (since the 8 cells are not independently distributed). We therefore have to correct for the number of biovolume units per detection unit to recalculate into actual detection unit counts (`N`). The `unit.size.txt` table was compiled by Birger Skjelbred for all identified taxa:

```
us <- read.table("data/unit.size.txt", header = TRUE, sep = "\t")

pp <- merge(pp, us)
pp$N <- ceiling(pp$Number / pp$Unit.size)

effort <- aggregate(pp$N, by = list(ID = pp$ID), sum)
summary(effort$x)
```

```
##    Min. 1st Qu.  Median    Mean 3rd Qu.    Max.
##   394.0   658.0   784.0   853.9  1015.0  2106.0
```

Number of counted detection units per sample varied from ca. 400 to > 2000. Which makes 400 a sensible target for rarefaction analysis.

Adjusting singletons for different subsample sizes

Standard phytoplankton counting procedures have been focused on estimation of biomass rather than biodiversity. This means that different taxa are counted over different subsamples (areas of the counting chamber) and/or at different magnifications. In other words, a count of 1 does not does not mean that there was only 1 unit in the sample, just in that particular subsample. Since single and double occurrences play a special role in some richnness estimators, we have a potential problem since all singletons in a sample may not be from the same subsample size.

The `Factor` field is inversely proportional to counted area, such that the smallest value of `Factor` will correspond to the largest counted area in a given sample. In other words, if we find the smallest value of `Factor` for each sample (`fmin`), then we can use this to adjust all counts in the same sample to the same subsample volume. By rounding upward (`ceiling()`), we ensure that the adjusted counts (`Nadj`) remain positive.

```
fmin <- aggregate(pp$Factor, by = list(Lake = pp$Lake), min)
names(fmin) <- c("Lake", "fmin")

pp <- merge(pp, fmin)
pp$Nadj <- ceiling(pp$N * pp$Factor / pp$fmin)

sum(pp$Nadj == 1) / dim(pp)[1]
```

```
## [1] 0.1155108
```

```
sum(pp$Nadj == 2) / dim(pp)[1]
```

```
## [1] 0.04943768
```

This adjustment hopefully has less bias in the number of singletons (11.6%) and doubletons (4.9%) than the original uncorrected counts.

Crosstabulating adjusted counts

We are now ready to use `tapply()` to crosstabulate the adjusted counts by sample (`ID`) and taxon (`Rubin.code`). Since missing values in the crosstabulation are true absences, we need to change them to zeros.

```
nt <- tapply(pp$Nadj, list(as.factor(pp$ID), pp$Rubin.code), sum)
nt[is.na(nt)] <- 0 # Replace NAs with true zeros
nt <- nt[, colSums(nt) > 0] # Remove empty taxa
dim(nt)
```

```
## [1]  73 395
```

Many of the 395 taxa are rare, and less than half the species are present in 5 lakes or more.

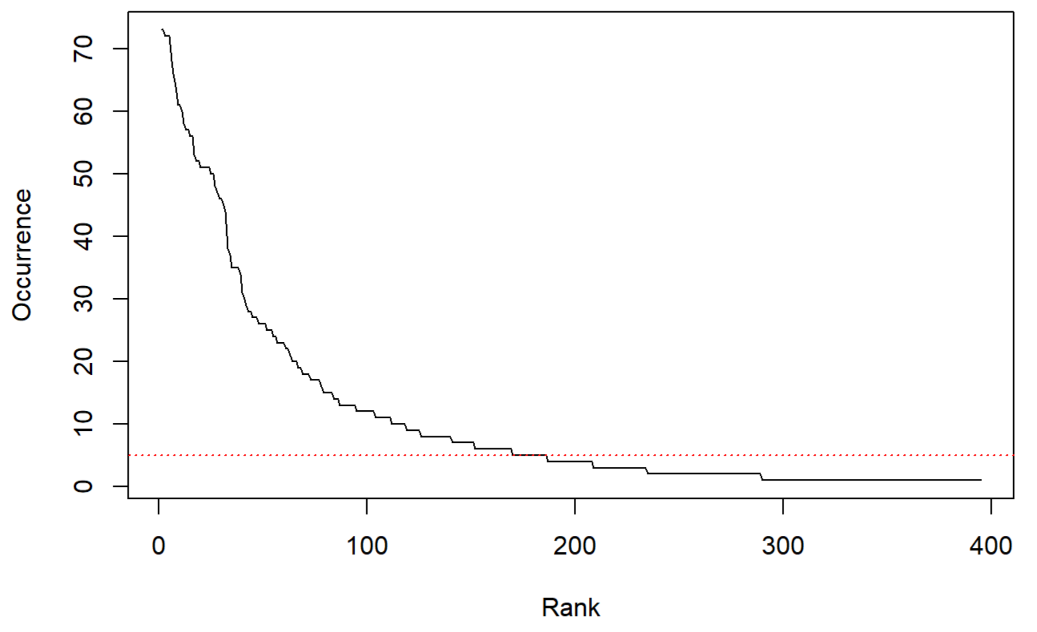

Fig. S2.1. Rank- occurrence curve for all phytoplankton taxa. Red dotted line marks occurrence in 5 lakes

Observed taxon richness

We will consider raw species richness calculated from all 395 taxa and also from the subset of 186 taxa present in more than 4 lakes

```
S <- data.frame(
  S.obs = rowSums(nt > 0),
  S.gt.4 = rowSums(nt[, colSums(nt > 0) > 4] > 0)
)
```

Extrapolated taxon richness

The `estimateR()` function from the vegan package estimates Chao and ACE richness from the subsampling-adjusted count data

```
s <- estimateR(nt) # Chao & ACE richness estimators
S$S.chao1 <- s["S.chao1", ]
S$S.ACE <- s["S.ACE", ]
```

Biovolume-rarefied richness

Rarefaction allows us to adjust richness estimates according to counting effort, by rarefying all counts to the sample with the least counting effort. Different taxa being counted at different resolutions and subsample sizes makes it difficult to use rarefaction directly on the adjusted counts, since rescaling for true singletons will inflate the counts of common taxa. Instead of a count-based resampling we therefore use a biovolume fraction-based rarefaction, as used by Özkan et al (2016).

For that, we first need to crosstabulate biovolumes by lake and taxon, and normalize to taxon contributions to relative biovolume contributions.

```
bv <- tapply(pp$Bio.vol, list(as.factor(pp$ID), pp$Rubin.code), sum)
bv[is.na(bv)] <- 0 # Replace NAs with true zeros
bv <- bv[, colSums(bv) > 0] # Remove empty taxa

bv.tot <- rowSums(bv)
pv <- sweep(bv, 1, bv.tot, "/")
```

The biovolume-based rarefaction consists of sampling taxa with replacement with probabilities equal to their relative biovolume fractions for each lake. We set number of samples equal to the smallest detection unit count (400)

```
N.rare <- 400

N.loc <- dim(pv)[1]
N.tax <- dim(pv)[2]

S.rare <- rep(NA, N.loc)
for (i in (1:N.loc)) {
  z <- sample((1:N.tax), N.rare, replace=TRUE, prob=pv[i, ])
  S.rare[i] <- sum(table(z) > 0)
}

S$S.rare <- S.rare
```

Genus richness

We can make a less operator-dependent and more robust richness measure by counting the number of genera instead of number of species. For this, we first need to crosstabulate the count data by `Genus` instead of taxon (`Rubin.code`), and then calculate genus richness as the sum of genus occurrences within sites

```
ng <- tapply(pp$Nadj, list(as.factor(pp$ID), pp$Genus), sum)
ng[is.na(ng)] <- 0  # change NAs to true absences

S$G.obs <- rowSums(ng > 0) # Genus richness
```

Information theory-based diversity indices

We also use the relative biovolume fractions to calculate information theoretical diversity indices (Shannon (H) and Simpson (D)) and from them, the first and second Renyi numbers (H1 = exp(H) and H2 = 1 / D)

```
p.log.p <- -pv * log(pv)
p.log.p[pv == 0] <- 0 # Since 0 * log(0) = 0, by definition!

Shannon.H  <- rowSums(p.log.p)
Simpson.D  <- rowSums(pv * pv)

S$H1 <- exp(Shannon.H)
S$H2 <- 1 / Simpson.D
```

Phytoplankton richness and diversity indicators

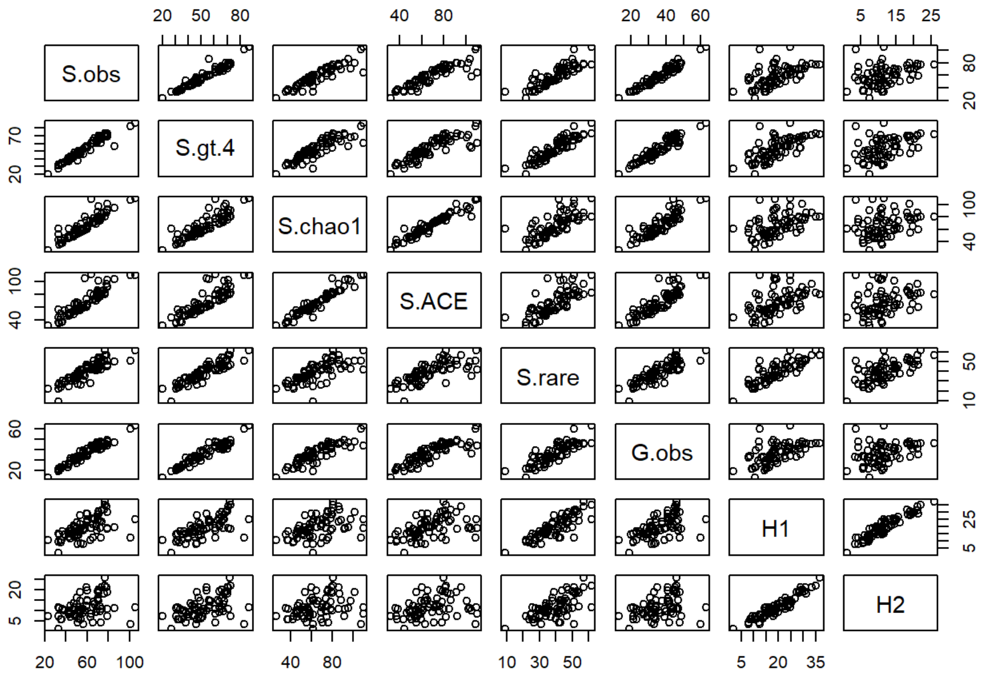

Fig. S2.2. Scatterplot matrix of 8 different phytoplankton richness and diversity indicators

```
m <- list(
  m.gt.4  = lm(S.gt.4 ~ S.obs, data = S),
  m.chao1 = lm(S.chao1 ~ S.obs, data = S),
  m.ACE   = lm(S.ACE ~ S.obs, data = S),
  m.rare  = lm(S.rare ~ S.obs, data = S),
  m.G.obs = lm(G.obs ~ S.obs, data = S),
  m.H1    = lm(H1 ~ S.obs, data = S),
  m.H2    = lm(H2 ~ S.obs, data = S)
)

R2 <- sapply(m, function(x) summary(x)$r.squared)
slope <- sapply(m, function(x) summary(x)$coefficients[2, 1])
se.slope <- sapply(m, function(x) summary(x)$coefficients[2, 2])
```

Table S2.1. Slopes with standard errors for regressions of alternative richness and diversity indicators against observed richness

	slope	se.slope	R2
m.gt.4	0.846	0.028	0.927
m.chao1	1.051	0.057	0.827
m.ACE	1.057	0.064	0.793
m.rare	0.561	0.038	0.756
m.G.obs	0.583	0.018	0.939
m.H1	0.263	0.044	0.338
m.H2	0.123	0.036	0.144

All the richness indices are strongly correlated with observed richness, while the correlations with information theoretical diversity indices (H1 and H2) are weaker. Taxon richness estimated from the taxa observed in at least 5 lakes is 85% $\pm$ 3% of observed richness, while genus richness is 58% $\pm$ 2% of taxon richness (1.7 taxa / genus). Extrapolated richness estimators (Chao1 and ACE) are both about 5% $\pm$ 5% higher than observed richness, while rarefied richness is on the average 58% $\pm$ 4% of observed richness. The “effective number of species” as indicated by the Renyi numbers (H1 and H2) are around 26% $\pm$ 4% and 12% $\pm$ 4% of observed richness, respectively. Since all richness and diversity indicators correlate reasonably well with observed richness (`S.obs`) it makes sense to focus on this particular indicator in the rest of this work.
