# Supplementary material for: Congruence, but no cascade—Pelagic biodiversity across three trophic levels in Nordic lakes
Source: Ecol Evol. 2020 Jul 17;10(15):8153–65. doi: 10.1002/ece3.6514 (PMC7417247; doi:10.1002/ece3.6514)
Supplement: Supplementary file 3 — Appendix S3 [file ECE3-10-8153-s003.pdf]

Suppl. 3 ordinations

Tom Andersen

2 april 2019

```
library(vegan)
```

Load data sets for environment (`env`), phytoplankton (`phyto`), zooplankton (`zoo`), and fish (`fish`), all extracted from text files located in the `/data` folder

```
source("data.0 env.R")
source("data.1 phyto.R")
source("data.2 zoo.R")
source("data.3 fish.R")
```

Phytoplankton ordination

Phytoplankton data consists of relative biovolumes of 208 taxa present in >3 of the 73 lakes. The NMDS ordination on square-root transformed relative biovolumes converged in <100 iteration with stress = 0.20. Ordinations with all 395 taxa or on just the 92 identified genera were very similar (data not shown)

```
phyto.freq <- colSums(phyto > 0)
phyto.rich <- rowSums(phyto > 0)

mds.phyto <- metaMDS(sqrt(phyto), trymax = 200, trace=0)
phyto.fit <- envfit(mds.phyto, env[, c(4:6, 10, 12, 13)])
```

Table S3.1. Environmental variables fitted to phytoplankton NMDS scores, with p-values based on 999 permutations

	NMDS1	NMDS2	R2	Pr(>R)
Latitude	-0.960	0.281	0.194	0.002
Longitude	0.535	0.845	0.422	0.001
Altitude	-0.652	0.758	0.181	0.002
log.Cond	0.814	-0.580	0.407	0.001
log.TOC	0.877	0.481	0.249	0.001
log.TP	0.889	-0.457	0.353	0.001

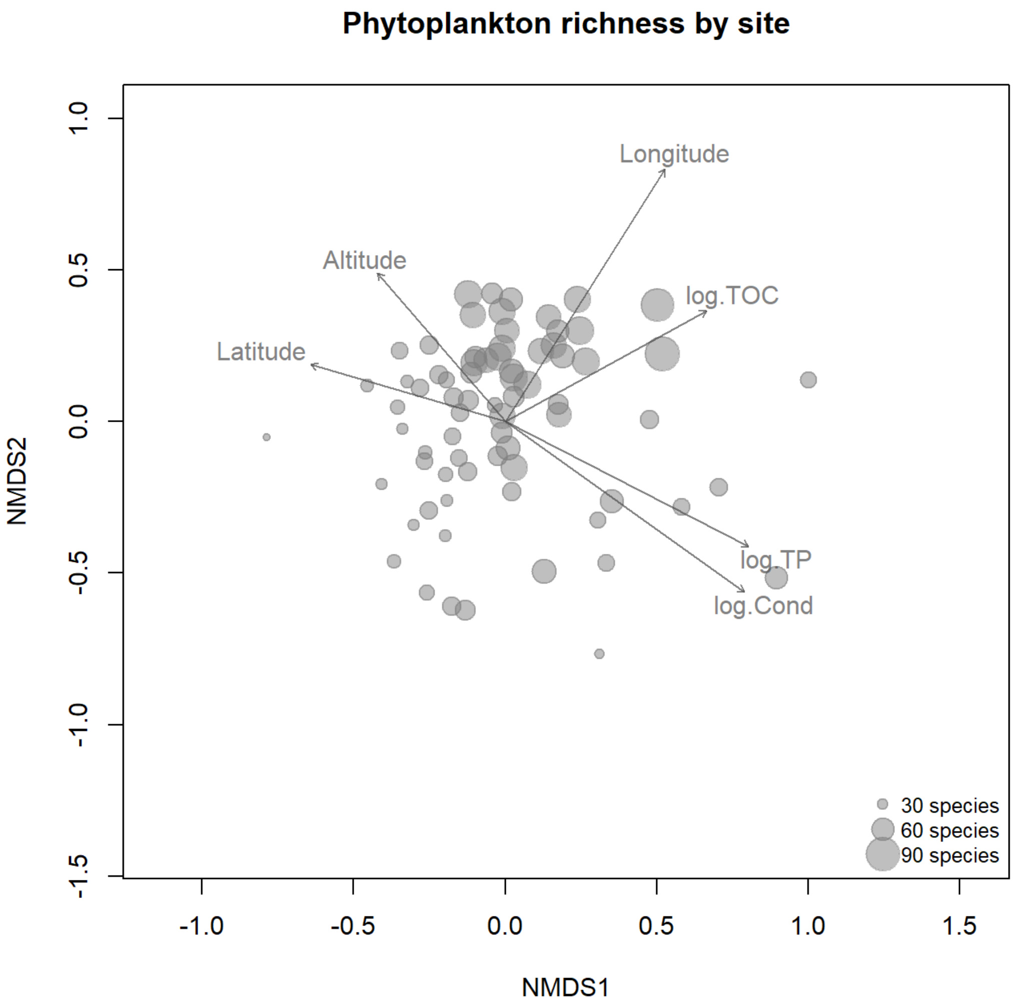

Figure S3.1. Site scores in phytoplankton NMDS ordination scaled by species richness, and overlaid fitted environmental variable vectors

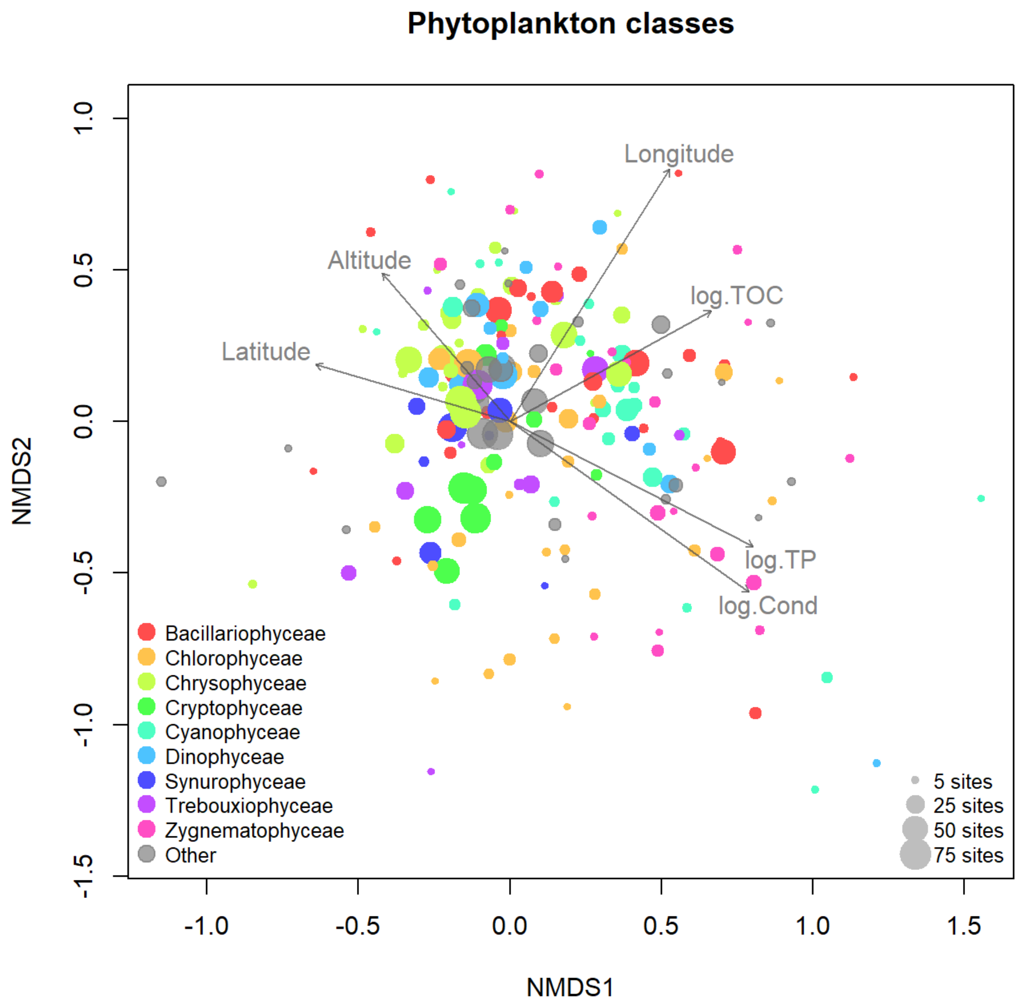

Figure S3.2. Phytoplankton NMDS species loadings scaled by species occurrence frequency and color coded by class, with overlaid fitted environmental variable vectors

Due to the larger number of taxa, we have left out species names in the phytoplankton ordination (Figure S3.2) while color coding individual species class level. Along the longitude / TOC vector there is a distinct taxon sorting with high loadings of the major Cryptophyceae taxa in the western, clear-water lakes and high loadings of diatom taxa (Bacillariophyceae) in the eastern, brown-water end of the gradient. Similarly, there is a tendency for Chrysophyceae dominance in the higher altitude / latitude lakes with increasing cyanobacteria loadings towards the higher productivity (TP / COND) end of the gradient.

Zooplankton ordination

NMDS ordinations using zooplankton abundance or presence/absence, on the full species inventory or the common ones encountered in the subsample, were all very similar (Procrustes correlations = 0.75 to 0.99, all with p = 0.001 based on 999 permutations). Based on the general similarity between zooplankton ordinations for different subsets, we chose to base the following analysis on relative abundances of the species encountered in the subsample.

Zooplankton data consists of relative abundances of 34 pelagic crustacean taxa encountered in the 73 lakes. NMDS ordinations converged in <100 iterations with stress = 0.19. Ordinations that also included littoral or rare species were very similar (data not shown)

```
zoo.freq <- colSums(zoo > 0)
zoo.rich <- rowSums(zoo > 0)

mds.zoo <- metaMDS(zoo, trymax=200, trace=0)
zoo.fit <- envfit(mds.zoo, env[, c(4:6, 10, 12, 13)])
```

Table S3.2. Environmental variables fitted to zooplankton NMDS scores, with p-values based on 999 permutations

	NMDS1	NMDS2	R2	Pr(>R)
Latitude	-0.521	0.853	0.210	0.001
Longitude	0.952	0.306	0.612	0.001
Altitude	-0.408	0.913	0.111	0.020

	NMDS1	NMDS2	R2	Pr(>R)
log.Cond	0.498	-0.867	0.356	0.001
log.TOC	0.975	-0.223	0.454	0.001
log.TP	0.806	-0.591	0.365	0.001

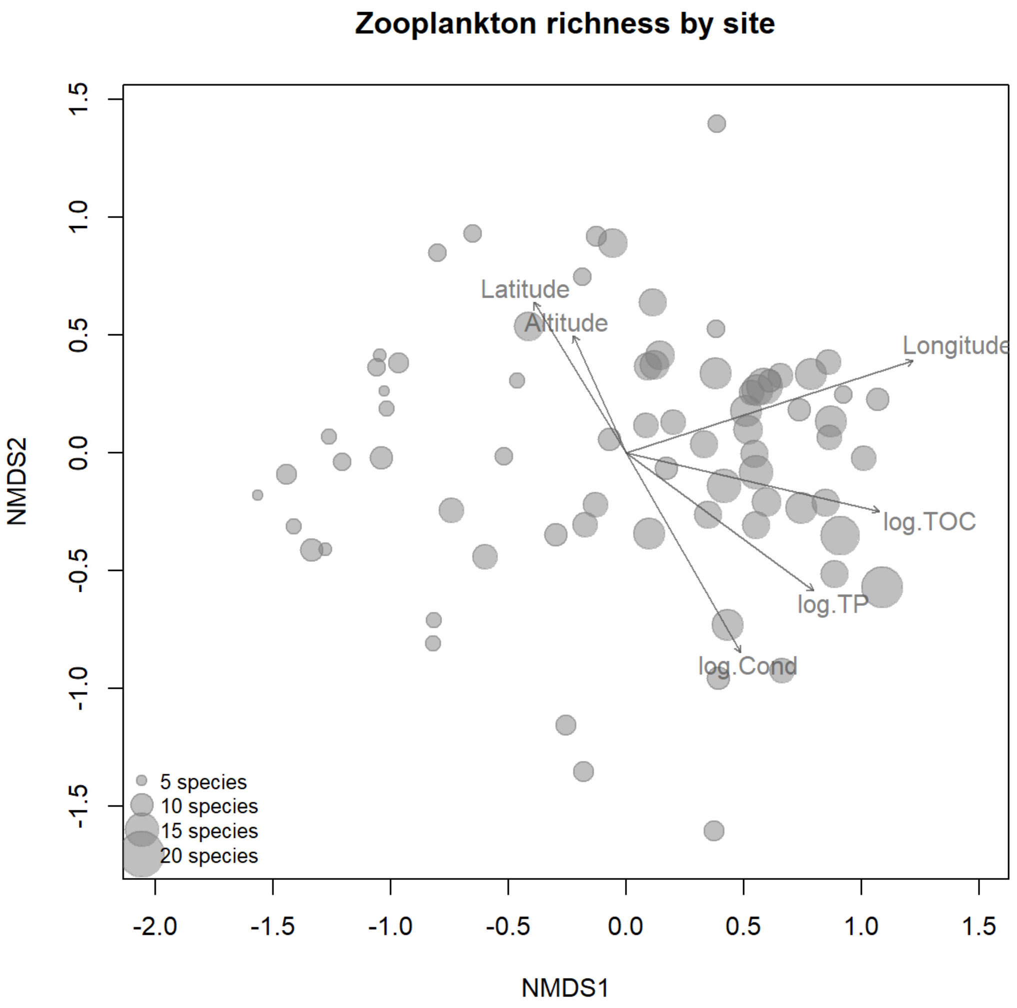

Figure S3.3. Site scores in zooplankton NMDS ordination scaled by species richness, and overlaid fitted environmental variable vectors

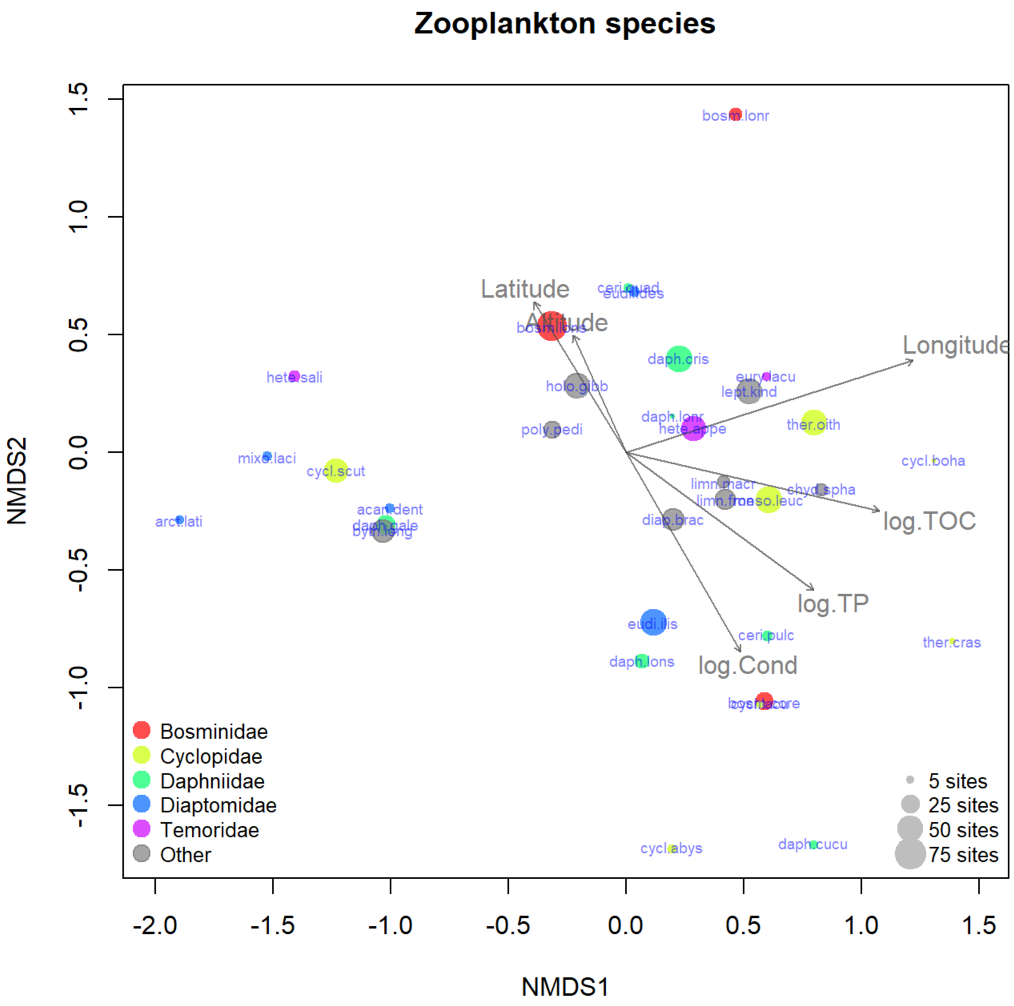

Figure S3.4. Zooplankton NMDS species loadings scaled by species occurrence frequency and color coded by family, with overlaid fitted environmental variable vectors

The zooplankton ordination (Fig. S3.4) shows high loadings for especially large-bodied calanoid copepods (Arctodiaptomus laticeps, Mixodiaptomus laciniatus, Heterocope saliens, Acanthodiaptomus denticornis) and cladocerans (Daphnia galeata, Bythotrephes longimanus) in the western part of the LON / TOC gradient. Along the productivity gradient there are distinct species replacements in the cladoceran genera Bosmina and Daphnia, with B. longispina and D. cristata being replaced by B. longirostris / coregoni and D. longispina / cucullata with decreasing altitude / latitude and increasing TP / COND. Two small and relatively rare cyclopoid copepods (Cyclops bohater and Thermocyclops crassus) are only found in the eastern-most lakes.

Fish ordination

Fish data consists of presence/absence of the 31 fish species registered in the 73 lakes. NMDS ordinations converged in <10 iterations with stress = 0.14.

```
fish.freq <- colSums(fish > 0)
fish.rich <- rowSums(fish > 0)

mds.fish <- metaMDS(fish, k=2, trymax=200, trace=0)
fish.fit <- envfit(mds.fish, env[, c(4:6, 10, 12, 13)])
```

Table S3.3. Environmental variables fitted to fish NMDS scores, with p-values based on 999 permutations

	NMDS1	NMDS2	R2	Pr(>R)
Latitude	0.255	0.967	0.080	0.050
Longitude	-0.988	-0.151	0.780	0.001
Altitude	-0.133	0.991	0.630	0.001
log.Cond	-0.204	-0.979	0.288	0.001
log.TOC	-0.998	-0.059	0.537	0.001

	NMDS1	NMDS2	R2	Pr(>R)
log.TP	-0.493	-0.870	0.278	0.001

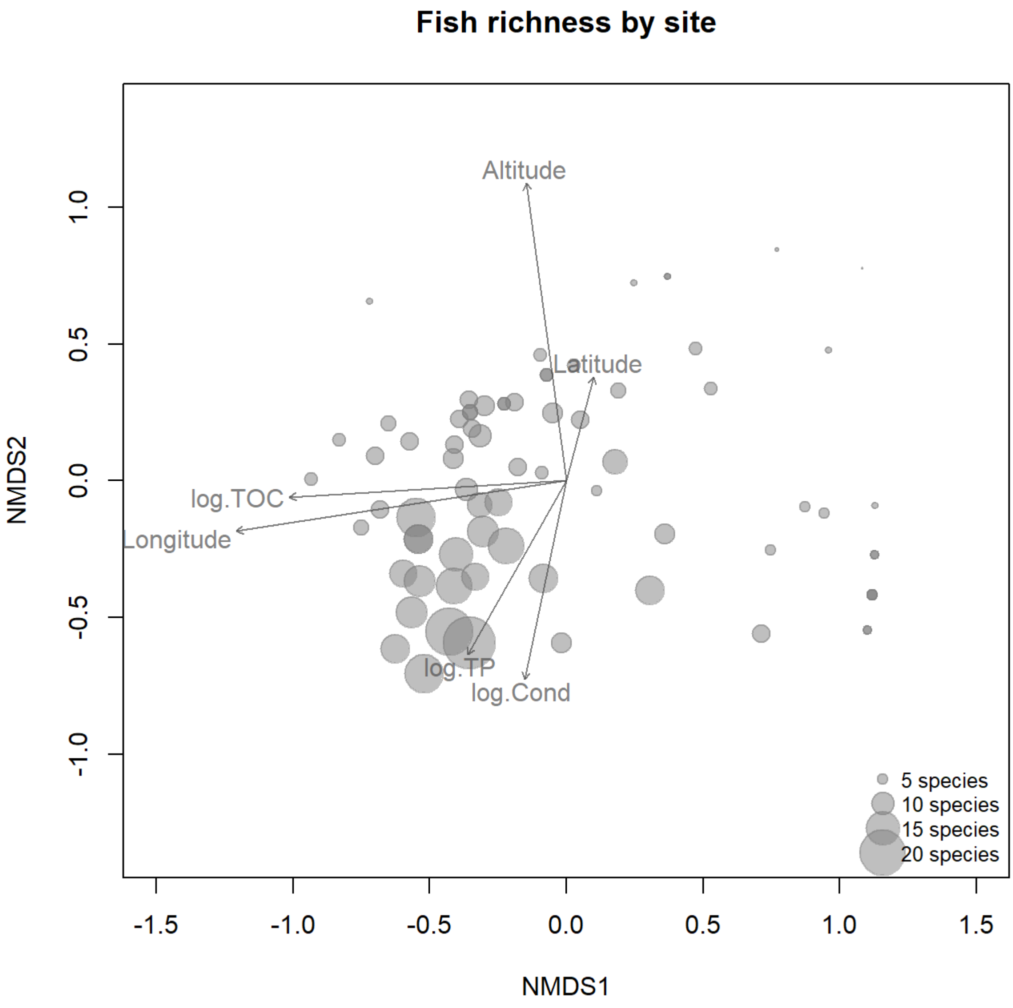

Figure S3.5. Site scores in fish NMDS ordination scaled by species richness, and overlaid fitted environmental variable vectors

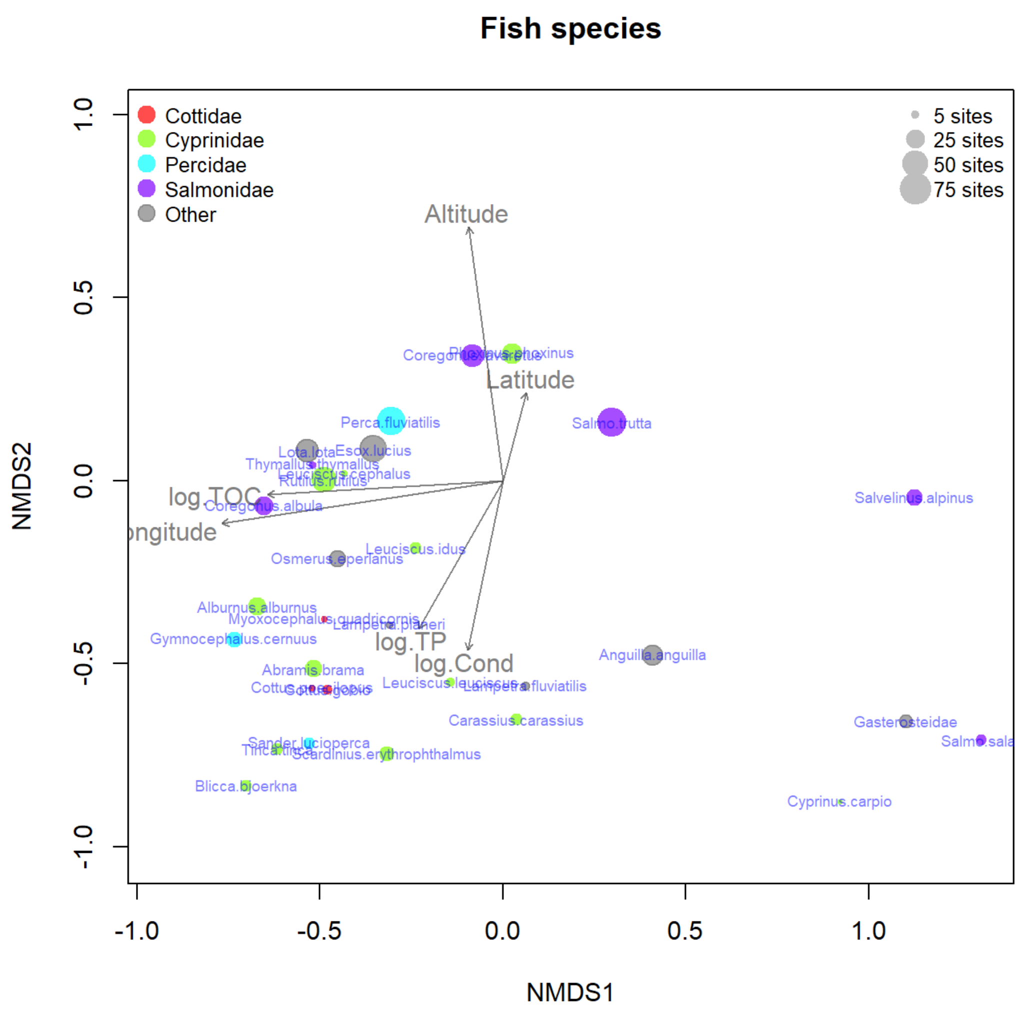

Figure S3.6. Fish NMDS species loadings scaled by species occurrence frequency and color coded by family, with overlaid fitted environmental variable vectors

The fish community ordination (Fig. S3.6) shows a clear separation between the ocean-side invaders (salmonids (Salmo trutta / salar and Salvelinus alpinus), sticklebacks (Gastrosteida), and European eel (Anguilla anguilla)) and the remainder of the fish species, especially cyprinids and percids, which are thought to have followed post-glacial invasion paths through the freshwater phases of the Baltic Sea. Notable exceptions to this pattern are carp (Cyprinus carpio) and minnow (Phoxinus phoxinus) which are expected to have been widely spread by humans. A number of cyprinids, such as silver and common bream (Blicca bjoerkna and Abramis brama), tench (Tinca tinca), and rudd (Scardinius erythropthalmus) have high loadings in the low latitude / altitude, high TP / COND lakes.
